# Electrochemistry, ion adsorption and dynamics in the double layer: a study of NaCl(aq) on graphite†

**DOI:** 10.1039/d1sc02289j

**Published:** 2021-07-14

**Authors:** Aaron R. Finney, Ian J. McPherson, Patrick R. Unwin, Matteo Salvalaglio

**Affiliations:** Thomas Young Centre and Department of Chemical Engineering, University College London London WC1E 7JE UK a.finney@ucl.ac.uk m.salvalaglio@ucl.ac.uk; Department of Chemistry, University of Warwick Coventry CV4 7AL UK

## Abstract

Graphite and related sp^2^ carbons are ubiquitous electrode materials with particular promise for use in *e.g.*, energy storage and desalination devices, but very little is known about the properties of the carbon–electrolyte double layer at technologically relevant concentrations. Here, the (electrified) graphite–NaCl(aq) interface was examined using constant chemical potential molecular dynamics (CμMD) simulations; this approach avoids ion depletion (due to surface adsorption) and maintains a constant concentration, electroneutral bulk solution beyond the surface. Specific Na^+^ adsorption at the graphite basal surface causes charging of the interface in the absence of an applied potential. At moderate bulk concentrations, this leads to accumulation of counter-ions in a diffuse layer to balance the effective surface charge, consistent with established models of the electrical double layer. Beyond ∼0.6 M, however, a combination of over-screening and ion crowding in the double layer results in alternating compact layers of charge density perpendicular to the interface. The transition to this regime is marked by an increasing double layer size and anomalous negative shifts to the potential of zero charge with incremental changes to the bulk concentration. Our observations are supported by changes to the position of the differential capacitance minimum measured by electrochemical impedance spectroscopy, and are explained in terms of the screening behaviour and asymmetric ion adsorption. Furthermore, a striking level of agreement between the differential capacitance from solution evaluated in simulations and measured in experiments allows us to critically assess electrochemical capacitance measurements which have previously been considered to report simply on the density of states of the graphite material at the potential of zero charge. Our work shows that the solution side of the double layer provides the more dominant contribution to the overall measured capacitance. Finally, ion crowding at the highest concentrations (beyond ∼5 M) leads to the formation of liquid-like NaCl clusters confined to highly non-ideal regions of the double layer, where ion diffusion is up to five times slower than in the bulk. The implications of changes to the speciation of ions on reactive events in the double layer are discussed.

## Abstract 2

CμMD reveals multi-layer electrolyte screening in the double layer beyond 0.6 M, which affects ion activities, speciation and mobility; asymmetric charge screening explains concentration dependent changes to electrochemical properties.

## Graphical abstract

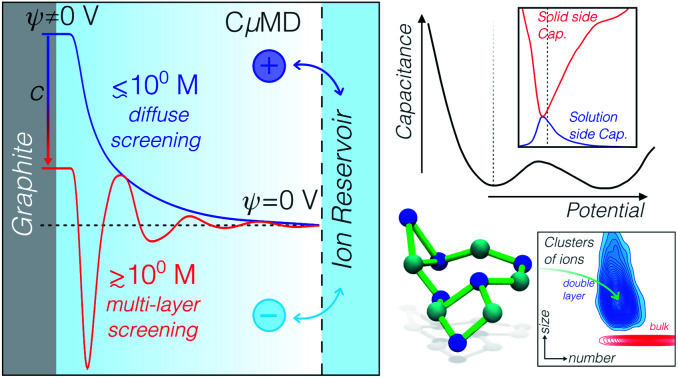

## 1 Introduction

Carbon–electrolyte interfaces are fundamental to the operation of many technological devices, particularly in the areas of energy storage (*e.g.*, supercapacitors), filtration and sensing.^1–7^ More generally, understanding chemical activity in the vicinity of interfaces is important for catalysis, corrosion and crystallisation.^8–12^ Graphite provides a model substrate to study such interfaces; current technological applications involving carbon materials *e.g.*, activated carbon, which offers a high surface-to-volume ratio for maximum ion adsorption, are often made up of more-or-less ordered microdomains of graphite.^13^ Improving the design of these materials, therefore, requires an understanding of the graphite–electrolyte interfacial structure and dynamics, where many details remain unresolved.^14^

It is at the interface between solids and liquid solutions where changes occur to the average structure and dynamics of charge carriers (*cf.* in the bulk) when the two phases come into contact, even in the absence of a potential bias. This results in the formation of the so-called double layer and an electric potential difference (drop) in this region. Early models of the double layer sought to explain the capacity for charge storage at the interface, which can be measured as the differential capacitance, *C*^d^.

One such Poisson–Boltzmann based model is the Gouy–Chapman–Stern (GCS) model (see ESI Section B for more details†) that predicts a compact layer of surface-charge compensating counter-ions adjacent to a charged, planar surface, followed by a diffuse solution layer enriched in counter-ions and depleted in co-ions.^15–17^ At low electrolyte concentrations and with low applied surface potentials, this model effectively predicts *C*^d^, in simulations^18^ and in systems containing metals,^19^ even if the simplified interfacial geometry it implies is unphysical for the electrode/electrolyte interface.^20^

The value of *C*^d^ measured at graphite is much lower than expected from consideration of GCS theory alone. This was rationalised based on the material's low electron density of states (DoS) at the Fermi level^21^ compared to metals, and the potential drop across the double layer was ascribed to both ion charge accumulation at the interface and space-charging (electron redistribution) within the solid.^22–24^ Assuming a GCS electrolyte structure, *C*^d^ was then used to estimate the electronic DoS of graphite.^23,24^

At moderate to high electrolyte concentrations—relevant to many technical applications—experimental observations suggest that the GCS model inadequately describes the solution-side structure of the double layer at graphite. For example, at 0.5 M and beyond, *C*^d^ depends upon the cation identity, and asymmetries in the *C*^d^–potential curves are found.^25–27^ This deviation is consistent with an observed inflection point in the screening length of NaCl(aq) solution as a function of concentration around 0.5 M from surface force balance experiments.^28^ These changes have been attributed to ion-specific adsorption,^25^ with possible partial charge transfer;^29^ however, without a detailed microscopic description of the interface, the origin of the variations in solution behaviour remain unclear.

Modifications to Poisson–Boltzmann-based models of the double layer were proposed to take into account ion correlations, specific adsorption and steric effects.^30–33^ Nonetheless, the complexity of the system favours the application of atomistic simulations to fully characterise the double layer structure that results from changes to bulk electrolyte concentrations and applied surface potentials. These methods allow for a molecular description of how the complex interplay between ion adsorption, solvation and speciation in the double layer, coupled with any changing mobilities for charge carriers, affects the electrochemical and thermodynamic properties of the interface.

Calculations at the level of density functional theory (DFT) with implicit solvents have provided significant insight into the nature of charge screening in the double layer.^29,34^ Molecular dynamics (MD) simulations adopting classical force fields, however, are the preferred tool to investigate the structure and dynamics of electrolytes in explicit solvents in contact with graphitic surfaces.^35–42^ Based on DFT calculations, classical pairwise interaction potentials were parameterised;^37–39^ these capture the polarisability of the solution and carbon (in the form of ion–π interactions) at the interface, which can play a significant role in structuring the double layer.^43,44^ Such classes of models further indicate asymmetric adsorption of ions, with cations likely to adsorb in preference to anions in the case of graphite. This contrasts with metal–electrolyte interfaces, where ion–π interactions are absent and anion adsorption likely dominates.^45^ The implications of these effects on the structure and dynamics of the double layer at graphite will be addressed in the present article by combining our own simulations and experimental measurements.

Constant chemical potential MD (CμMD)^46^ simulations were performed to explore the double layer in the (electrified) graphite–NaCl(aq) system. CμMD mimics open boundary conditions; thus, maintaining a constant thermodynamic driving force for ion adsorption at graphite, and conserving electroneutral solutions beyond the double layer. With this approach, we are able to relate the spatial extent of the double layer to the nature of charge screening in this region. We explain changes to the electrochemical properties of the double layer by relating the screening of charge to the potential difference across this region. In addition, we obtain a detailed description of the local (electro)chemical potential, speciation and mobility of ions orthogonal to the surface. The results indicate a significant departure from ideal solution behaviour in regions confined to the double layer even at moderate levels of NaCl(aq) concentration in the bulk. Our work provides much needed molecular-level insight into the structure and dynamics of electrolyte solutions in contact with carbon surfaces over a wide range of concentrations.

## 2 Results and discussion

### 2.1 The structure of NaCl(aq) solutions at the graphite surface

Simulation cells were prepared where a graphite slab, comprising eight graphene layers, was positioned at the centre of the simulation cell *x* axis in contact with NaCl(aq), such that the system was symmetrical about *x* = 0. All methodological details are provided in ESI Section A.† CμMD simulations were performed using the GROMACS (v 2018.6) MD package^47^ with the Plumed (v 2.5) Plugin^48^ for a range of fixed bulk solution concentrations: *c*^b^_NaCl_ = 0.23–1.05 M and 1.2–9.2 M in two system set-ups. The upper limit here significantly exceeds the solubility of NaCl in water, and would not be possible to prepare experimentally, but is instructive to study computationally. The nanosecond timescales associated with MD give rise to vanishingly low probabilities for crystal nucleation and allow the metastable solution state to be investigated. Within minimal fluctuations, the CμMD method successfully maintained the bulk concentration of cations and anions beyond the interface (see ESI Section C†).

#### Concentration profiles orthogonal to the surface

**Fig. 1:**
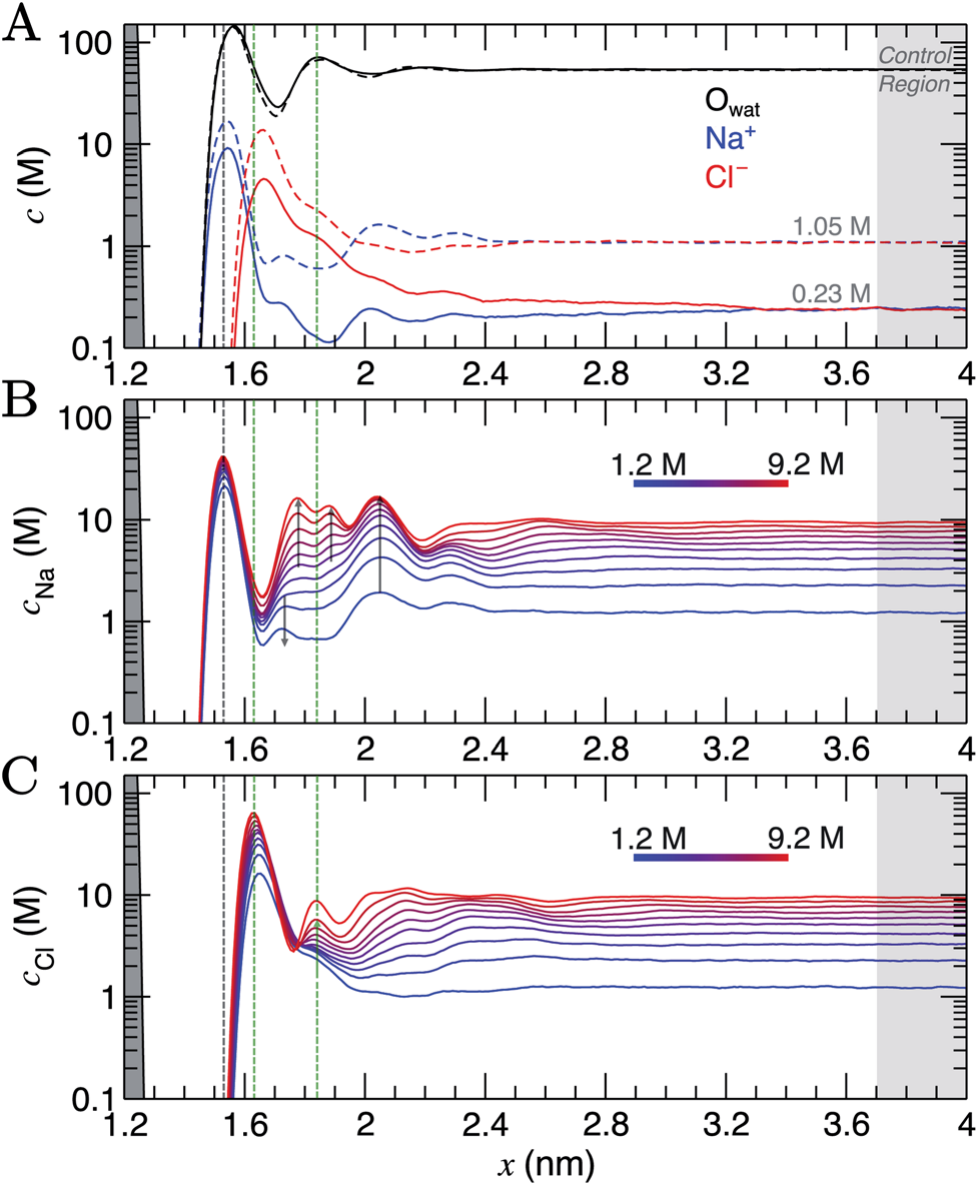
Ion and water molar concentration (*c*) profiles from CμMD simulations as a function of *x*: the distance from the centre of the simulation cell. The graphite carbon surface (with zero applied charge) is shown by the grey peak on the left of the *x* axis. (A) Provides Na^+^ (blue), Cl^−^ (red) and water oxygen (O_wat_; black) concentrations when the concentration of ions in the bulk is 0.23 M (solid line) and 1.05 M (dashed line). (B and C) Provide the cation (top panel) and anion (bottom panel) concentrations as a function of *x* in simulations targeting the higher end of the total concentration range. The colour scale here indicates the mean molarity of ions in the bulk. The shaded dashed lines mark the maximum in the first sodium peak (grey) and first two chloride peaks (green) at the highest bulk concentration. Arrows are provided to highlight the changes in the profiles as ion concentrations in the bulk are increased. A 0.3 nm excluded region separates the edge of the graphite basal plane from the ionic solution; this is due to atom centres being used to calculate the concentration profiles.

Concentrations as a function of *x* for species in the solution phase are provided in Fig. 1 and also in full in Fig. S5 and S6.† Preferential adsorption of Na^+^ was observed at the graphite surface over the entire concentration range sampled, in line with the predicted order of ion adsorption energies using the adopted force field.^37^ Solvated Na^+^ ions directly coordinate to graphite, shown by the narrow peak at *x* = 1.5 nm in Fig. 1A. Maximum concentrations exceed those in the bulk by approximately two orders of magnitude even at the lowest *c*^b^_NaCl_ (0.23 M). An adjacent layer of Cl^−^ ions was observed, separated by 0.1 nm at the peak concentrations, and in line with other simulations.^49^ The Cl^−^ concentration in this layer also significantly exceeds the bulk value, although its broader width highlights a diffuse ordering of the anion. Considering the adsorbed layer of Na^+^ to represent an effective surface charge density, this picture of charge screening is qualitatively consistent with the predictions of the GCS model; with the Cl^−^ diffuse region of the double layer representing the counter-ion charge in solution. However, no obvious boundary between this region and a diffuse layer is apparent in the concentration profiles, and any binary assignment of surface-bound states neglects the complexity of the dynamic adsorption in the first ion layers.

As the bulk concentration of ions is raised (see Fig. S5†), a clear departure from the above screening behaviour is observed. Around *c*^b^_NaCl_ = 0.5 M, the Cl^−^ peak narrows and a second, diffuse layer of cations around *x* = 2 nm emerges that exceeds bulk ion concentrations. Fig. 1A highlights that at *c*^b^_NaCl_ = 1.05 M, the concentration of cations exceeds that of anions at *x* = 2–2.4 nm, and a hierarchical ordering of ions with opposing charge is apparent. A more compact double layer region is evident at the highest concentrations (see Fig. 1B and C, with the complete data set available in Fig. S6†). In the range *c*^b^_NaCl_ = 1–9 M, a shift in the position of the Cl^−^ first peak by Δ*x* ≈ −0.03 nm highlights a contraction of the first ion layers, and the diffuse Cl^−^ peak at 1.2 M was resolved into two clear peaks (with a second peak emerging at *x* = 1.83 nm at around 6 M bulk ion concentrations). This is concomitant with a shifting of the second Na^+^ peak away from the graphite surface, which ultimately splits into a rather diffuse doublet peak which confines the Cl^−^ layer.

The crowded structure at higher concentrations is reminiscent of the double layer in molten LiCl at planar electrodes under the constraint of a constant applied potential.^50^ Indeed, in double-layer capacitors containing ionic liquids, steric crowding at the electrode is a common feature,^51,52^ which has been confirmed by atomic force microscopy (AFM).^53,54^ The similar response of the double layer structure to changes in bulk concentrations and applied surface potentials was identified in simulations over 40 years ago.^18^ As well as the increasingly non-monotonic concentration profiles (shown clearly in Fig. S7A† on increasing *c*^b^_NaCl_), a shift in the ratio of maximum Na^+^ : Cl^−^ concentrations occurs around 3 M due to the narrowing of the second Cl^−^ peak (see Fig. S7B†) which represents a significant departure from the double layer structure at the lowest concentrations.

Perturbations to the solvent structure were apparent, mainly due to the presence of graphite. Three peaks are observed (see Fig. 1A) in the water concentration profiles at the lower end of the bulk ion concentration range simulated. Solvent layers are separated by 0.3 nm; this distance was determined for water at clean graphite surfaces in a recent study combining simulations with AFM measurements.^55^ Only limited ordering of the orientation of water molecules perpendicular to the graphite surface was observed. This is highlighted in Fig. S5,† which shows that the peaks for water O and H atoms are approximately at the same position in *x*. Maximum values of *c*_Cl_ at the interface tended to be found where the densities of water oxygen atoms are close to a minimum. Additional ion layers at the highest concentrations induce additional complexity to the water structure in the double layer (see Fig. S6†).

#### Extent of the double layer

**Fig. 2:**
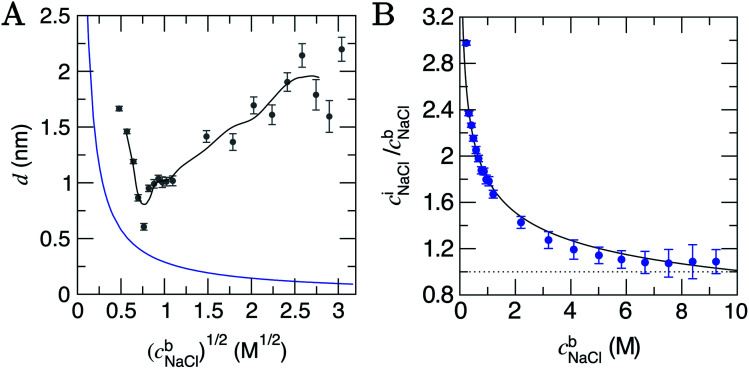
(A) The depth of the double layer region, *d*, in CμMD simulations with varying scaled bulk ion concentrations, (*c*^b^). The grey points provide the size of the double layer in *x*, with error bars indicating uncertainties of one standard deviation in the data. The black line provides a moving average for the data. Note that the minimum value of *x* for the solution phase (with reference to Fig. 1) was taken to be the mid-point between the carbon surface and the minimum value in ion concentration profiles: *x* ≈ 1.35 nm. Also shown in the figure is the blue curve which is the Debye length (see ESI Section B†). (B) Provides the normalised, mean interface ion concentrations (*c*^i^_NaCl_) in a 1.5 nm solution region close to the graphite surface *vs.* the bulk concentration of ions. The black line is a fit to the data with functional form 1.8(*c*^b^_NaCl_)^−0.25^ and error bars show uncertainties of one standard deviation.

The edge of the double layer region on approach to the surface was marked by the position where solutions deviate from electroneutrality; hence, 〈*c*_Na_(*x*)〉 ≠ 〈*c*_Cl_(*x*)〉 (where angular brackets indicate the mean concentrations in 0.5 nm moving windows in *x*). The size of the double layer from this position to the minimum in *x* for the solution phase is provided in Fig. 2A; this indicates that the double layer contracts as the ionic strength in the bulk solution initially increases, reaching a minimum around (*c*^b^_NaCl_)^1/2^ = 0.76 M^1/2^ (*c*^b^_NaCl_ ≈ 0.6 M). Beyond this bulk concentration, the double layer size increases, plateauing around 2 nm above (*c*^b^_NaCl_)^1/2^ = 2.4 M^1/2^, with some noise in the data. Overall, using this composition measure, the double layer size is 0.6–2.2 nm. The bulk electrolyte concentration marking a transition in the double layer structure (0.6 M) and the size of the double layer, within the concentration range sampled here, are in very close agreement with the results from surface force balance measurements which determine the screening length in NaCl(aq) solutions between parallel plates.^28^

The Debye length (*κ*^−1^) is the characteristic length over which the electrostatic effect of a charge carrier in solution decays, and is proportional to 1/(*c*^b^)^1/2^. This is derived from a linearised Poisson–Boltzmann equation (see Section B† for details), and is assumed to accurately determine the size of the double layer at low electrolyte concentrations. Fig. 2A indicates that *κ*^−1^ decreases monotonically as *c*^b^_NaCl_ increases. At relatively low concentrations, one can reconcile the decreasing double layer size from simulations by considering that increasing charge densities close to the surface will lead to a less diffuse double layer as contraction of the layers occurs. At high concentrations, however, the ionic crowding near the surface induces further perturbations to the solution away from the interface, and the double layer size increases with *c*^b^_NaCl_. A theoretical framework to predict the ‘capacitive compactness’ of the double layer was recently presented;^56^ this indicates the dependency of the size of the interfacial region upon the ion valency.

It is instructive to consider the size of the interface region where the solvent structure is perturbed (*cf.* the bulk), which turns out to be independent of *c*^b^_NaCl_ over the entire concentration range sampled. ESI Section D† details these measurements which indicate an interface region that is 1.4 ± 0.3 nm in size, that is approximately 3–5 water layers from the graphite surface. The structuring of water at planar interfaces appears to be rather insensitive to the electrolyte concentration and, for most practical purposes, the substrate material and surface contamination.^55^

#### Mean ion concentrations in the double layer

NaCl concentrations at the interface (*c*^i^_NaCl_) can be measured by integrating (*c*_Na_(*x*)*c*_Cl_(*x*))^1/2^ in regions of the profiles in Fig. 1. To ensure a fair comparison between different cases of bulk concentration, a 1.5 nm solution region closest to the graphite surface was integrated, and the *c*^i^_NaCl_ normalised by *c*^b^_NaCl_ are provided in Fig. 2B. The plot shows a rapid decay in *c*^i^_NaCl_/*c*^b^_NaCl_ on increasing *c*^b^_NaCl_, with concentrations at the interface converging to those in the bulk when *c*^b^_NaCl_ > ∼6 M.

At the lowest *c*^b^_NaCl_, the concentrations of ions at the interface are three times greater than those in the bulk and the decay in the relative interfacial concentrations is proportional to (*c*^b^_NaCl_)^−0.25^ (see Fig. 2B). While finite ion size effects clearly play a role in the local ion concentrations in the double layer, the total concentrations of ions in this region vary continuously with bulk concentration and can therefore be predicted without the need for simulations at specific concentrations. It is important to note that the total concentrations of cations and anions over the entire double layer region are equal, as shown in Fig. S8,† and that significant ordering at the highest bulk concentrations means that, locally, ion concentrations can significantly exceed the bulk (see Fig. S7A†).

#### Electrical properties of the double layer

Even at uncharged graphite surfaces, asymmetries in the adsorption of ions leads to deviations from local electroneutrality, as shown by fluctuations in the solution charge density, *ρ*, as a function of *x*. Following the Poisson equation,1
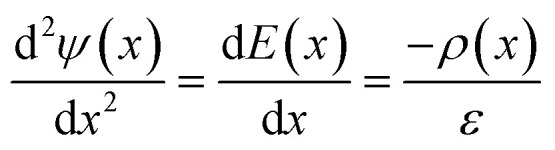
this leads to varying electric fields, *E*, and electric potential, *ψ* orthogonal to the surface. In the above equation, *ε* is the permittivity of the medium (*ε* = *ε*_0_*ε*_r_, where *ε*_0_ and *ε*_r_ refer to the permittivity of vacuum and the relative permittivity, respectively). Fig. S9† provides these quantities over the full concentration range. These indicate that, at the limit of large *x*, *E*(*x*) and *ψ*(*x*) converge to zero, corresponding to the solution bulk.

**Fig. 3:**
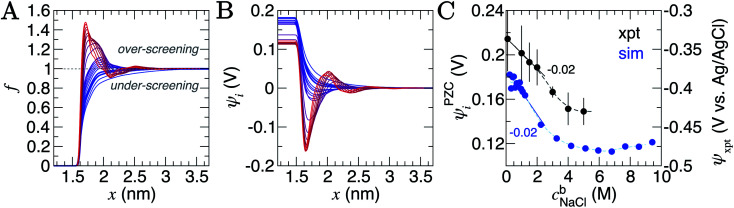
Charge screening in the double layer. (A) Provides the ion charge screening factor, *f*, as a function of distance, *x*, from the graphite surface. (B) Provides the electric potential, *ψ*, calculated from ion charge distributions in the same region. The colour scale from blue to red in (A) and (B) indicates increasing bulk ion concentrations across the entire concentration range: 0.2–9.2 M, as listed in Table S2 of ESI.† (C) Provides the potential difference across the interface region (*i.e.* the PZC) as a function of *c*^b^_NaCl_. The simulation data, taken from the difference in *ψ*_i_ in panel (B), are shown by the blue circles (the left *y* axis scale apply to these data only), and measurements from electrochemical experiments are provided by the black circles (the right *y* axis scale applies here). Statistical uncertainties of one standard deviation in the data are shown by the error bars. The dashed lines provide a moving average of the data and the solid lines are a linear fit to the data when *c*^b^_NaCl_ < 3 M, the gradients for which are provided.

A screening factor, *f*, can be defined as,2
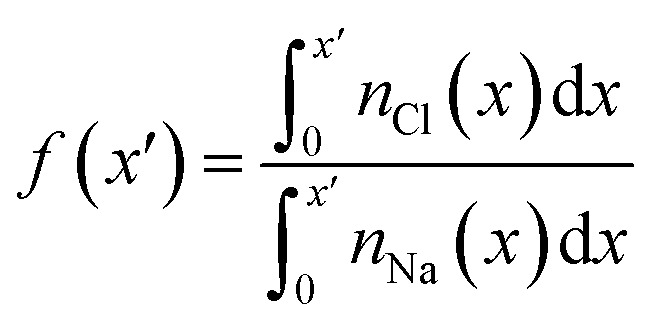
with *n* indicating ion number densities; *n*_Na_ appears in the denominator as cations consistently adsorb in the first solution layer next to the substrate. Fig. 3A provides *f*(*x*) for the entire concentration range sampled. At the lowest *c*^b^_NaCl_, *f*(*x*) increases smoothly and converges to one in the solution bulk, consistent with screening by a diffuse anion layer. As *c*^b^_NaCl_ increases beyond around 0.5 M, and the compensating anion charge layer becomes more compact, over-screening of the cation charge occurs and *f*(*x*) > 1. Over-screening in molten salts is a phenomenon that has been known for some time.^57^ In ionic liquids at electrified interfaces, over-screening was suggested as a possible control on the electrochemical kinetics at the interface.^58^

The electric potential in *x* due to the charge distribution of ions (*ψ*_i_) can be calculated using eqn (1) and (2), noting that *ρ*_i_(*x*) = *e*(*n*_Na_(*x*) − *n*_Cl_(*x*)), where *e* is the elementary charge:3
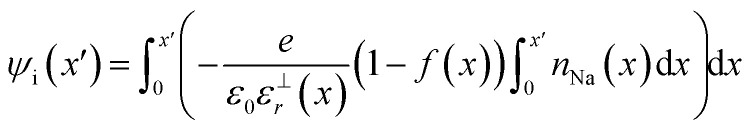


The relative permittivity of the solution medium as a function of *x* (*ε*^⊥^_r_(*x*)) is affected by the proximity of interfaces^59–62^ and ion concentrations.^63^ A recent simulation study from Olivieri *et al.*^64^ indicated that truncation of the long range water molecule dipole moment correlations due to the presence of the substrate and the electric field anisotropy at the interface leads to increasing *ε*^⊥^_r_ away from the surface. We adopted this model for the near-linear increase of *ε*^⊥^_r_, from a value of around 10 in the first solvent layer up to 71 (the bulk value of *ε*_r_ for the extended simple point charge water model^65^ in our simulations) over *x* ≈ 4 nm, as described in ESI† Section E—where we also discuss alternative models and their implications for the calculated *ψ*_i_(*x*) values.

The *ψ*_i_(*x*) curves calculated using eqn (3) are provided in Fig. 3B. When *c*^b^_NaCl_ ≳ 0.5 M, the sign of *ψ*_i_ alternates due to the crowding of ions in the double layer. The value of *ψ*_i_ at the graphite surface (with zero applied charge) is called the potential of zero charge (PZC): *ψ*^PZC^_i_. This potential difference is shown in Fig. 3C as a function of the bulk concentration. It is clear that the effect of increasing *c*^b^_NaCl_ is to decrease *ψ*^PZC^_i_ with a −0.020 ± 0.003 V M^−1^ gradient at moderate bulk concentrations. An inflection point is observed when *c*^b^_NaCl_ ≈ 6 M, where further increases in bulk ion concentrations result in positive changes to *ψ*^PZC^_i_.

The slope, (d*ψ*^PZC^_i_/d*c*^b^_NaCl_)_*T*,*σ*_ is related to the so-called Esin–Markov coefficient.^66^ This is one of the few conventional means to experimentally assess the extent of specific adsorption, and would seem to provide an ideal way to relate the simulations to experiments measuring *C*^d^. Often, *C*^d^ data are recorded at widely spaced potentials, limiting the accuracy with which the minimum in *C*^d^—often taken to represent PZC—is known.^25,27^ We therefore measured *C*^d^ of freshly exfoliated HOPG as a function of potential over a range of concentrations, with 10 mV potential resolution, and examined the minimum in *C*^d^ as a proxy for the PZC (*ψ*^xpt^; see Section A2†). With this fine potential resolution, the *C*^d^–*ψ*^xpt^ curve displays two minima within 300 mV; the global minimum becomes deeper and shifts to more negative potentials with increasing concentration (Fig. S2†). Note that this feature remains visible at 50 mV potential resolution (Fig. S3†), but would be lost at lower resolutions, often reported in the literature.^22,23,25,27^

Computational models considering the effect of asymmetric ion adsorption indicate a shift to the PZC on incremental changes to bulk solute concentration that follows the sign of the preferentially adsorbing ion.^50^ However, these often do not consider the situation of electrolyte solutions in which both cations and anions have favourable, but varying strength of interactions with the surface. Experimental studies sometimes attribute *C*^d^ values solely to the adsorption of ions whose sign is opposite to the sign of the potential change relative to the *C*^d^ minimum.^25^ This is a rather simple interpretation under conditions where the bulk concentration is far beyond the levels where alternating layers of charge emerge in the double later structure. The change in the value of *ψ*^xpt^ identified at the *C*^d^ minimum on increasing *c*^b^_NaCl_ (see Section A2† and Fig. 3C) is in good agreement with the mean change in *ψ*^PZC^_i_(*c*^b^) found from our simulations, with a moderate negative gradient (−0.02 V M^−1^) at lower concentrations. This contrasts with the linear shift as a function of ln (*c*^b^_NaCl_) seen on Hg electrodes.^67^ The agreement provides considerable support to the simulation results.

The implication of eqn (3) is that increasing levels of ion screening, with the same underlying cation density distribution, results in positive changes to *ψ*^PZC^_i_. Conversely, if the screening of charges in the double layer is unchanging and the bulk concentration increases, then *ψ*^PZC^_i_ becomes more negative. In our simulations, Fig. S11† shows that at the limit of large *x*, ∫(1 − *f*(*x*)) decreases (due to increased levels of screening in a less diffuse counter-ion charge cloud) and, combined with increasing *c*^b^_NaCl_, the relatively large negative gradient, d*ψ*^PZC^_i_/d*c*^b^_NaCl_, at the lowest concentrations reduces on increasing *c*^b^_NaCl_. At the highest concentrations, however, the ordering of ions leads to a small positive change in ∫(1 − *f*(*x*)) on increasing *c*^b^_NaCl_. This is sufficient to change the sign of d*ψ*^PZC^_i_/d*c*^b^_NaCl_. It follows that, for small increases to *c*^b^_NaCl_, the resulting response to the PZC can inform about the structure of the double layer.

### 2.2 Ion activities in the double layer

The excess density of ions in the double layer has implications for the chemistry of this region. It is helpful to evaluate, therefore, contributions to the electrochemical potential, *μ̃*_i_, of ions, i, as a function of *x*:

4 *μ̃*_i_(*x*) = *μ*^0^ + *β*^−1^ln *a*_i_(*x*) + *z*_i_*eψ*(*x*)

here, *μ*^0^ is a reference chemical potential; *a*_i_(*x*) and *z*_i_ are the activity and valency of species i, respectively; and, *β* = 1/*k*_B_*T*, where *k*_B_ and *T* are the Boltzmann constant and temperature, respectively. The total *μ̃* for NaCl(aq) in *x* can be written as,

5 *μ̃*(*x*) = *μ*^0^ + *β*^−1^ln *a*_±_(*x*) + (2*ω*(*x*) − 1)*eψ*(*x*)

where *ω* is the fraction of cations and *a*_±_(*x*) is a position-dependent average activity. We define *a*_±_(*x*) = *a*_Na_(*x*)^*ω*(*x*)^*a*_Cl_(*x*)^1−*ω*(*x*)^, which converges to the mean ion activity, *a*^b^_±_ = (*a*^b^_Na_*a*^b^_Cl_)^1/2^, in the electroneutral bulk solution where *ω*(*x*) = 0.5.^68^ Eqn (4) and (5) therefore reduce to the standard equations for the chemical potential (*μ*) in a bulk homogeneous solution where *ψ*(*x*) = 0 in the absence of an external electric potential. At equilibrium, *a*_±_(*x*) and *ψ*(*x*) are stationary, and the electrochemical potentials across the double layer and in the extended solution are equal:

6 *β*^−1^ln *a*_±_(*x*) + (2*ω*(*x*) − 1)*eψ*(*x*) = *β*^−1^ln *a*^b^_±_

Based on fits to the chemical potentials of ions explicitly calculated in their own simulations and from other *in silico* studies,^69,70^ Zimmerman *et al.*^71,72^ provided an analytical model to calculate the mean chemical potential of solvated ions using the adopted force field:

7 *μ*_NaCl_ = *μ*^0^_NaCl_ + 2*β*^−1^ln *b*_NaCl_ + 2*β*^−1^ln *γ*_±_

where *b* is the mean molality in units of mol kg^−1^, and *γ*_±_ is the mean activity coefficient for ions; therefore, *a*_±_ = *b*_NaCl_*γ*_±_, and,8
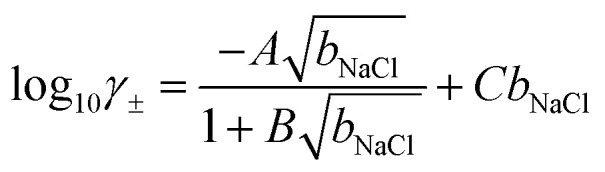
where *A* = 0.568 m^−1/2^, *B* = 1.17769 m^−1/2^ and *C* = 0.177157 m^−1^ (where m = mol kg^−1^). Importantly, this model allows us to calculate ion activities at molalities far beyond the equilibrium saturation level of 3.7 mol kg^−1^.^69,70,73–76^ Values of ln *γ*_±_ and *μ̃*_NaCl_ calculated using the above model are provided for the range of *b*_NaCl_ in Fig. S12.†

From CμMD simulations, the NaCl molality as a function of *x* is calculated from atom density profiles according to (*n*_Na_(*x*)*n*_Cl_(*x*))^0.5^/(0.018*n*_wat_(*x*)). Molalities in the bulk (*b*^b^_NaCl_(*x*)) were calculated from averages in the molality profiles in stable regions far from the interface. *b*^b^_NaCl_(*x*) were substituted into eqn (8) and (7) to calculate (*μ*_NaCl_ − *μ*^0^_NaCl_)/2 which equals the right hand side of eqn (6). Note that this approach assumes equal contribution of cations and anions to the mean ion electrochemical potential. The CμMD simulation technique used here ensures accurate estimates of the bulk chemical potential of ions—within the adopted model in eqn (7)—where cation and anion concentrations must be uniformly equal within a small uncertainty.

**Fig. 4:**
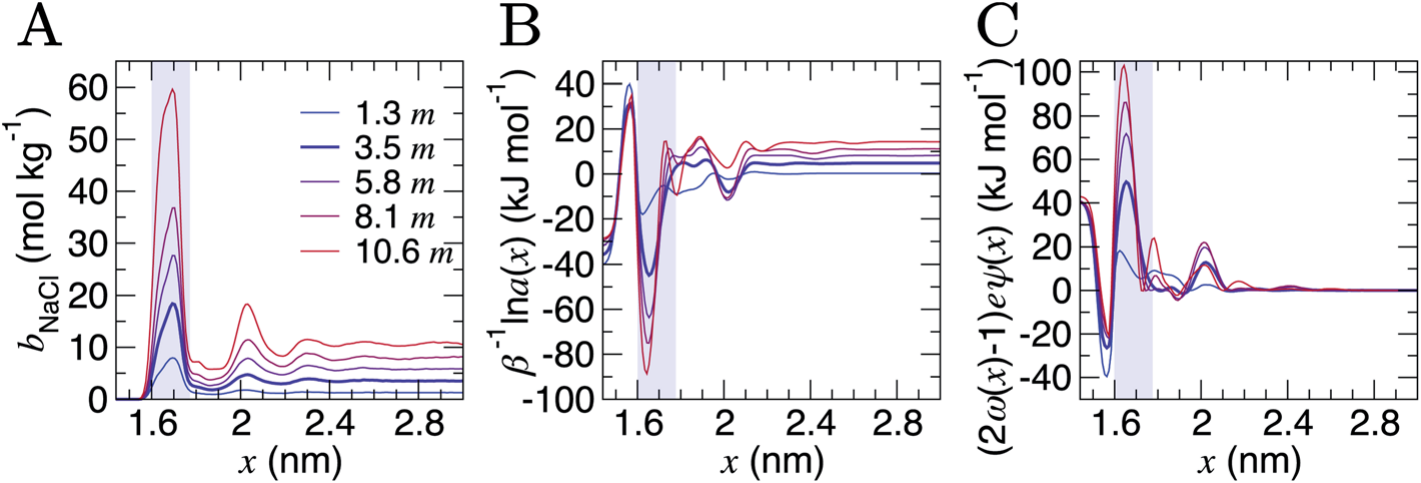
Contributions to the electrochemical potential of electrolytes in the double layer region. (A) NaCl molalities (*b*^b^_NaCl_) when the bulk mean molalities were as shown in the legend in units of *m* (mol kg^−1^). (B) The contribution to the mean ion molal electrochemical potential (*μ̃*) from the first term in eqn (6). (C) The contribution of the second term in eqn (6) to *μ̃*. The curve for the case when *b*^b^_NaCl_ = 3.5 mol kg^−1^ is highlighted by the bold line, and the shaded region indicates the position of the maximum peak in *b*^b^_NaCl_.

Analyses were performed using the density profiles in Fig. 1, with Fig. 4A providing the NaCl molality profiles for five systems. These reach a maximum at *x* = 1.6–1.7 nm: the position close to the minimum following the first peak in cation concentration profiles. An increasing peak around *x* = 2 nm matches with the increase in cation concentrations in this region at high concentrations. Counter-intuitively, the region in *x* around the maximum *b*^b^_NaCl_(*x*) corresponds to a minimum in *β*^−1^ln  *a*_±_(*x*) (see the shaded region Fig. 4B). This is due to a maximum in (2*ω*(*x*) − 1)*eψ*(*x*) as shown in Fig. 4C. The fact that at *x* = 1.63 nm, *ω* and *ψ* are at a minimum, results in the large positive contribution to *μ̃* from this term in eqn (6). Beyond around 1 nm from the graphite surface, we find that the contribution of (2*ω*(*x*) − 1)*eψ*(*x*) to *μ̃* is zero, and in this region *μ̃* reduces to *μ*.

An important implication of the above observation is that the local ion molality (or concentration) at interfaces is not a good proxy for the electrochemical potential of ions. In systems where interfaces and extended liquid phases are in equilibrium, this is usually not problematic, due to the equality *μ̃* = *μ*^b^ across the boundary layer and into the bulk solution. Although any partial charge transfer of surface-bound ions should be considered.^29,77^ In some surface-driven processes at equilibrium, knowledge of the interfacial structure might still be essential to predict outcomes. For example, in processes like salt precipitation, the rates for nucleation are affected by the kinetic factors associated with the supply of ions to growing crystalline embryos.^12^ Increased ion molalities close to the surface (being around five times the levels of the bulk, when the bulk molality equals the equilibrium saturation level of 3.7 mol kg^−1^) likely mitigates the barriers to these processes. In non-equilibrium processes, knowledge of both the local molality of solute species and the electric potential is essential to determine *μ̃*.

### 2.3 Ion correlations and diffusion

Simulations at the atomic level are perfectly suited to provide details regarding the collective arrangement and motion of ions in the liquid near the graphite surface. In this section, we characterise the species which emerge in the double layer at uncharged graphite surfaces and their diffusion.

#### Ion speciation

We investigate ion speciation by calculating the average first shell coordination number, *N*_i*–*j_, between atoms i and j described in ESI Section A.† The average coordination numbers, *N*_Na–Cl_ = 0.02 ± 0.02; *N*_Na–Ow_ = 5.89 ± 0.05 and *N*_Cl–Ow_ = 7.23 ± 0.07 were calculated for a 1 M NaCl(aq) bulk solution simulated for 10 ns. The structure of the solvated ions agrees well with other simulation and experimental studies.^78^ A majority of ions form solvent shared and solvent separated ion-pairs in the bulk (represented by the peaks at *r* ≈ 0.5 and 0.7 nm in Fig. S13†).

Fig. S14† provides the average *N* as a function of *x* evaluated using Gaussian kernel (with 0.03 nm bandwidth) probability densities. These indicate that the average *N*_Na–Ow_(*x*) decreases by a value of one from the lowest to highest sampled concentrations in the bulk regions as *N*_Na–Cl_(*x*) changes from zero to one, with these changes becoming significant when *c*^b^_NaCl_ ≳ 1 M. Interestingly, *N*_Cl–Ow_(*x*) is far less sensitive to changes in *c*^b^_NaCl_, which remain around the value identified in the bulk at 1 M over then entire bulk concentration range within the model. The *N*_Na–Cl_(*x*) coordination profiles in Fig. S14† A indicate a greater number of directly coordinated ions beyond the position of maximum densities in *x* for the first ion layers. Essentially, the increased coordination occurs in regions of the double layer where there is a high density of both cations and anions. The maximum in *N*_Na–Cl_(*x*) shifts to smaller values of *x* and an additional peak emerges at *x* ∼ 2.2 nm as crowding in the double layer increases. The features of these profiles roughly correspond to the profiles of *b*^b^_NaCl_(*x*) in Fig. 4A, which is a good indication that the association of ions in the double layer is due to increased ion densities.

**Fig. 5:**
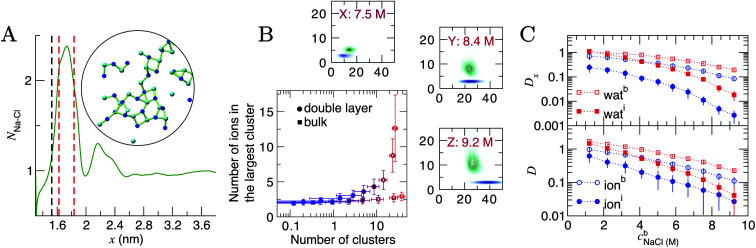
Ion assembly and diffusion in the double layer. (A) Average Na–Cl first-sphere coordination number (*N*_Na–Cl_) as a function of *x* calculated from a CμMD simulation where *c*^b^_NaCl_ = 9.2 M. The black and red dashed lines indicate the maximum first Na and first two Cl densities in the concentration profiles for Na and Cl highlighted in Fig. 1. Inset is a configuration of a particularly large ionic network identified within the red dashed lines in *x*. The image is projected onto the *yz* plane; blue and cyan spheres represent Na^+^ and Cl^−^ ions and the green lines highlight ions that are directly coordinated. (B) Ion clusters observed in the bulk and double layer regions of simulations. The blue → red colour scale in the main plot indicates increasing *c*^b^_NaCl_ from 1.2 to 9.2 M. The surrounding panels show purple and green probability densities in the same 2D space for clusters in the bulk and in the double layer, respectively, at the highest bulk concentrations shown by the legend. (C) Diffusion coefficients, *D*, for ions and water in 0.4 nm regions at the interface and in the bulk region of CμMD simulations (indicated by the subscripts i and b in the legends). The top panel provides the *x* component of D. Data have been scaled by 1 × 10^−5^ cm^2^ s^−1^ and error bars show uncertainties of one standard deviation.

At the highest levels of *c*^b^_NaCl_, *N*_Na–Cl_ > 2 at the maximum positioned at *x* = 1.75 nm, as shown in Fig. 5A. Concomitant changes occur to *N*_Na–Ow_(*x*) in this region, with an additional minimum at *x* = 1.6 nm. The exceedingly high anion concentrations here affect the ability for water to fully solvate cations. The increased Na–Cl coordination is due to the formation of many contact ion pairs that dynamically (dis)associate on the timescales of the simulations, leading to extended liquid-like networks of the type identified in the inset of Fig. 5A. These structures begin to emerge at the graphite surface in significant numbers when the concentration of ions in the bulk exceeded approximately 5 M (*i.e.*, beyond the nominal equilibrium saturation level for this force field). The networks are reminiscent of other liquid-like ionic networks identified in simulation studies,^79^ which were suggested as precursors to crystalline phases.^80,81^

To analyse these structures further, we performed cluster analyses using the method of Tribello *et al.*^82^ (see ESI Section A† for details). Fig. 5B indicates that the effect of increasing *c*^b^_NaCl_(*x*) is to increase the number of ion clusters (defined as species containing more than two ions in direct contact) in the bulk and within the double layer. In the bulk, these clusters contain a maximum of three ions. In the double layer, however, the clusters contain more than ten ions at the highest *c*^b^_NaCl_(*x*), with a wide distribution in the size of the largest clusters due to the rapid time evolution of ion–ion correlations. Both the size and geometry of the networks rapidly changed over several nanoseconds of simulation and exchange of ions with the surrounding solution occurred. Similar liquid-like NaCl clusters were identified in simulations beyond the limit of solution stability (15 mol kg^−1^) in the bulk, where a change in the mechanism for salt precipitation occurs.^83^ Some experimental studies posit the existence of NaCl clusters even at moderate saturation levels.^84^ Further studies are now needed to explore the role that liquid-like clusters play on the nucleation of NaCl at interfaces.

#### Diffusion in solution

The diffusion coefficients, *D*, for ions were measured using the Einstein relation, described in detail in ESI Section A.† For reference, the average *D* for ions measured in simulations of bulk of 1 M NaCl(aq) was 1.14 ± 0.05 × 10^−5^ cm^2^ s^−1^.

*D* and *D*_*x*_ as a function of *x* are provided in Fig. S15; † these indicate that the surface decreases the diffusion of ions and water molecules within the double layer. The decrease in both *D*(*x*) and *D*_*x*_(*x*) on approach to the substrate is monotonic; hence, the diffusion coefficients closest to the graphite surface and in the bulk region have been plotted as a function of *c*^b^_NaCl_ in Fig. 5C for simulations sampling the higher end of the entire concentration range. The values of *D* were found to decay following an approximately exponential trend: *D* = *D*_0_e^−*λc*b^, where *λ* is the so-called decay constant. In the bulk, *D*_0_ were 1.344 and 2.396 × 10^−5^ cm^2^ s^−1^ and *λ* were −0.236 and −0.386 for ions and water, respectively. A more negative *λ* for water indicates that increasing ion concentrations retards the mobility of the solvent molecules moreso than solute ions. At the interface, however, *λ* for ions was −0.335 (*D*_0_ = 0.819 × 10^−5^ cm^2^ s^−1^): more negative than for water (*λ* = −0.221; *D*_0_ = 2.248 × 10^−5^ cm^2^ s^−1^), which is most likely due to the increased concentration of ions in this region and the changes to the speciation of ions, discussed above.

Around 1 M, *D*^i^/*D*^b^ = 0.64 and 0.87 for ions and water, respectively (where *D*^i^ and *D*^b^ are the diffusion coefficients close to the graphite surface and in the bulk). At the highest concentrations sampled, *D*^i^/*D*^b^ = 0.24 and 0.18 (for ions and water, respectively). The arresting of particle mobilities is largely due to decreased diffusion perpendicular to the interface. The top panel in Fig. 5 provides the *x* component of *D* for water and ions at the interface and in the bulk. At 1 M, *D*^i^_*x*_/*D*^b^_*x*_ = 0.35 for ions and this reduces to 0.03 at the maximum bulk concentration. This reflects the high charge densities close to the graphite surface. In contrast, *D*^i^_*x*_ for water molecules is unchanged compared to *D*^b^_*x*_ at the lowest concentration, but beyond 9 M, *D*^i^_*x*_/*D*^b^_*x*_ = 0.09, and the high salinity interface retards the mobility of water molecules in *x* nearly as significantly as for ions.

Ion transport properties in the double layer are often assumed to match with those in the bulk, *e.g.*, when calculating *ζ*-potentials using electrokinetic flow apparatus. Diffusion coefficients for ions in solution near the graphite surface on the order 1 × 10^−7^ to 1 × 10^−5^ cm^2^ s^−1^ indicate an increased viscosity in the double layer caused by the changing solution densities in this region. While direct coordination of cations to the graphite was evident, no specific surface-site binding was identified, and diffusion was particularly limited perpendicular to the graphite surface. This picture is arguably consistent with the idea of a ‘dynamic Stern layer’.^85,86^ However, this term is unhelpful,^20^ failing to recognise the dynamic equilibrium between ions in the first and adjacent solution layers. No clear boundary (slipping plane) between the diffusion of ions in a specifically adsorbed layer at the surface and in the diffuse region can be identified from *D* or *D*_*x*_ in Fig. S15.† We refer the reader to a recent monograph by Döpke and Hartkamp,^86^ where these effects are discussed in the context of electrokinetic phenomena.

### 2.4 Graphite with applied electric charge

To consider the effect of applied electric fields, charges were uniformly distributed to the outermost carbon atoms at the graphite basal plane, discussed in detail in ESI Section A.† Negatively and positively charged surfaces were simulated with charge densities, *σ*, in the range |*σ*| = 0.004–1 *e* nm^2^ (see ESI Table S2†). Our approach neglects the electronic response of the graphite to charging the material, which must be considered when comparing to experiments. This is reasonable, considering that DFT calculations indicate that applying an electric field to graphite induces equal and opposite net excess *σ*, centered close to the edges of a graphite slab, perpendicular to the field direction.^21^

The concentration of ions beyond the double layer was maintained in CμMD simulations (see Fig. S16†) where *c*^b^_NaCl_ = 1.05 ± 0.03 M. Assuming Poisson–Boltzmann behaviour, Grahame's equation (see eqn (13) in ESI Section B†) provides a direct relationship between (the effective) *σ* and the potential change across the double layer, Δ*ψ*. Even within the monotonic regime, Poisson–Boltzmann approximations can fail to accurately predict *σ*.^86–88^ These models are, therefore, unhelpful, particularly to determine the interfacial properties of NaCl(aq) on graphite at technologically-relevant electrolyte concentrations, as discussed below.

#### Structural asymmetries in the double layer

**Fig. 6:**
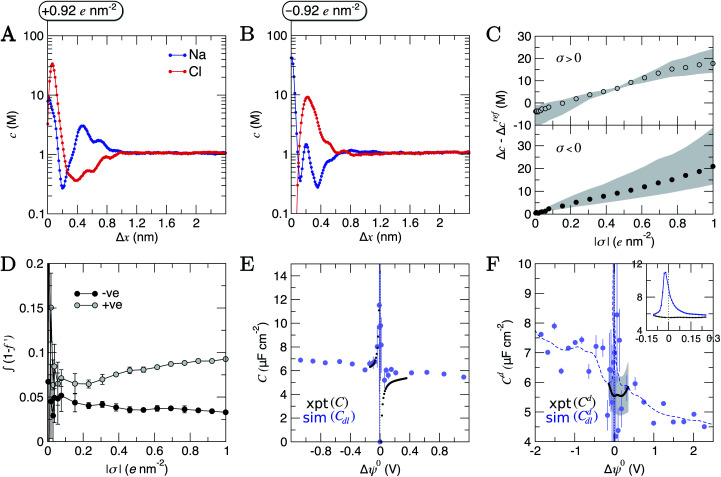
The effects of applied surface charges on the double layer when *c*^b^_NaCl_ ∼ 1 M. (A and B) Provide ion concentrations as a function of distance from the electrode (positioned at *x* = 0) measured in CμMD simulations when |*σ*| = ±0.92*e* nm^−2^, where *σ* is the surface charge density. (C) Provides the difference in the maximum ion concentrations (Δ*c*) at the electrode as a function of *σ*. Equivalent concentration differences, from distributions when no surface charge is applied (Δ*c*^ref^), were first subtracted, and shaded areas show uncertainties in the MD data due to changes using a smoothing window of 0.2 ± 0.1 nm. (D) Shows the integrals of 1 − *f*′, where *f*′ is the electrode screening factor, calculated for all atoms in the double layer region in solutions. (E) Provides the capacitance (*C*) as a function of the potential difference across the double layer after subtracting the potential of zero charge (Δ*ψ*^0^). Experimental data are calculated from the integral of the differential capacitance (*C*^d^) data in panel F for the 1 M case. (F) Provides the measured *C*^d^ from experiments and the evaluated double layer capacitance from simulations (*C*^d^_dl_) as a function of Δ*ψ*^0^. The dashed blue line is a moving average over three data points (in blue) at positive/negative Δ*ψ*^0^. This is shown more clearly in the inset of (F) over the range of potential differences used to measure *C*^d^ (also inset and shown by the black data points). Where shown, error bars highlight the standard error of the mean from multiple trajectory window analyses (otherwise statistical uncertainties are of the size or smaller than the data points), with the grey region in (F) showing the uncertainty in the mean *C*^d^ from three repeat experiments. In (E) and (F), data have been truncated for ease of comparison between simulations and experiments.

Ion number densities as a function of *x* in Fig. S17† show that when increasingly positive surface charges are applied, Cl^−^ ions were pulled closer to the graphite surface in a less diffuse anion layer. Concomitantly, the maximum density in the first cation layer decreased and was shifted further away from the surface. Despite this, a more compact double layer emerges due to increased cation densities in a second cation layer, shown most clearly at high values of *σ* in Fig. 6A. When the surface charge was made incrementally negative, the first two cation layers both increased in density and peaks in *n*_Na_(*x*) appear sharper. The maximum in the anion density decreased and was shifted away from the graphite, although the anion layer remained diffuse, with the density of the tails in the distribution actually increasing (see Fig. 6B and S17†).

Structural changes were observed in the solvent layers at the interface; these were particularly significant when *σ* was large, in line with simulations elsewhere studying electrified planar interfaces.^89^ Fig. S18† shows that when *σ* was large and positive, water oxygen atoms were pulled closer to the surface and peaks in *n*_Hw_(*x*) appear sharper. More interestingly, when *σ* was more negative than approximately −0.5 *e* nm^2^, a restructuring of water molecules was apparent at the interface, with a splitting of the first peak in *n*_Hw_(*x*), as solvent molecules arrange their hydrogen atoms towards and away from the graphite surface.

Changes to the excess ion number densities (*n*_i_ −*n*^ref^_i_, where *n*^ref^_i_ are the densities when *σ* = 0) as a function of *σ* are reported in Fig. S19.† These highlight that the double layer undergoes an asymmetric enrichment of charge-balancing counter-ions and depletion of co-ions. The asymmetries can be quantitatively evaluated by considering the excess counter-ion concentration at the charged graphite surface. This was done by taking the difference in ion concentrations, Δ*c* = *c*_A_ − *c*_B_ (where A is the counter-ion type and B is co-ion), from the maximum in concentration profiles where Δ*x* < 1.5 nm in the MD data. Fig. 6C shows that Δ*c* increases monotonically from a value of zero when *σ* = 0; this is qualitatively consistent with the predictions of the GCS model. At the positively charged surface, Δ*c* is negative when *σ* is small. The repulsion of cations in the first ion layer (beyond the electrode) leads to a small decrease in the maximum anion concentrations in the first anion layer and a small increase in the maximum concentrations in the second cation layer beyond the surface (see Fig. S19†). These observations are beyond the predictions of simple mean-field models and are due to the complex interplay of charge screening and volumetric constraints due to finite ion size effects at intermediate-high bulk concentrations.

#### Electrode charge screening

Asymmetries in the shape of the excess solution charge densities (defined as *ρ*^0^(*x*) = *ρ*(*x*) − *ρ*^ref^(*x*), where *ρ*^ref^ is the *σ* = 0 case, reported in Fig. S20†) appear in the first ion layer, with a doublet peak emerging at the negatively charged surface. Despite this, the features of the resulting *E*^0^(*x*) and *ψ*^0^(*x*) curves (calculated using the Poisson equation, and also provided in Fig. S20†) are largely symmetrical when comparing the oppositely charged surfaces. Differences occur in the amplitudes of the fluctuations in these curves. Fig. S22A† highlights a greater change to the electric potential across the double layer region, Δ*ψ*^0^, in response to applying positive charges (*cf.* negative charges) at the graphite surface. The potential difference diverges when |*σ*| = 0.1 *e* nm^2^.

To further understand the above divergence, we calculated an electrode screening factor:9
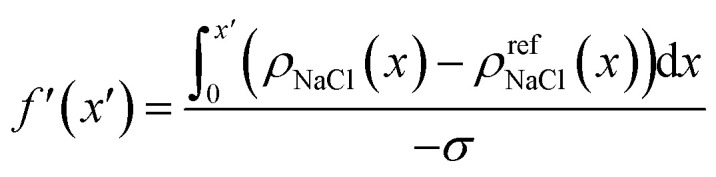


Fig. S21† shows that, considering only ions in solution, over-screening occurs at the positively charged surface due to the high density of anions in the first solution layer at the highest *σ*. *f*′ then decays until it converges to a constant value far from the graphite surface. Small changes to *σ* affect the maximum in *f*′(*x*) and, therefore, the gradient in the screening profiles as *f*′ converge to their bulk values. On the negatively charged surface, however, there is an initial over-screening that is compensated by charges in an adjacent solution layer, leading to a minimum in *f*′ around 1.6 nm. We calculated 
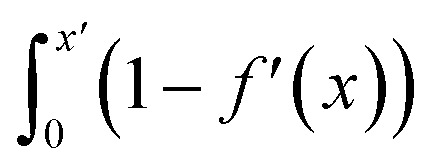
 (after ensuring that all *f*′ profiles converge to a value of one). The resulting curves at the highest values of applied potential are provided in Fig. S21.† A positive (negative) shift in the converged value of 
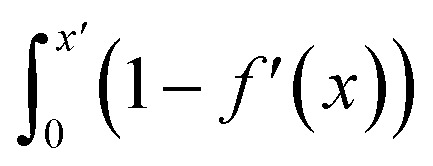
 is found at the positively (negatively) charged graphite surface. This is consistent with a more diffuse screening of the positively charged surface. When all solution atoms were included in the analyses, the same trends were observed, as highlighted by Fig. 6D; although the data here are noisy due to fluctuations in the water density profiles, particularly for small values of |*σ*|. These changes correlate with the changes to |Δ*ψ*^0^|, presented in panel D of Fig. S22A.†

The asymmetric accumulation of ions manifests in a greater capacity, *C* = *σ*/Δ*ψ*^0^, for the surface to store ionic charge when negative potentials are applied. Fig. 6E shows that maximum and minimum *C* occur at small negative and positive values of Δ*ψ*^0^. This is in very good agreement with the experimental *C* values (also show in Fig. 6E) calculated using the integral of *C*^d^(Δ*ψ*^0^) measured for the HOPG–NaCl(aq) system when *c*^b^_NaCl_ = 1 M (described in ESI A†). The asymptotic behaviour of *C*(Δ*ψ*^0^) is opposite to the trends at positive/negative potential observed in simulation studies of molten LiCl at atomically flat electrodes; however, these electrodes are not related to any specific material.^50^ When Δ*ψ*^0^ was highly negative, *C* is up to 2 μF cm^−2^ greater than when Δ*ψ*^0^ was large and positive.

Despite the close agreement between the simulated and experimentally determined values of *C*, the differential capacitance data (*C*^d^ = d*σ*/dΔ*ψ*^0^; provided in Fig. 6F) show deviations around Δ*ψ*^0^ = 0. Here, the mean simulated differential capacitance reaches a maximum around 11 μF cm^−2^ (shown inset of Fig. 6F, with the average over the full range of potential difference sampled in the experiments being 6.1 ± 1.2 μF cm^−2^ from simulations). Our simulations, however, only evaluate the contribution to the series of differential capacitance, 1/*C*^d^ = 1/*C*^d^_dl_ + 1/*C*^d^_q_, associated with the double layer response to the applied charge (*C*^d^_dl_); they neglect any contribution to *C*^d^ due to the (re)distribution of the graphite electron density of states associated to charging (*C*^d^_q_), which was postulated in early studies to dominate *C*^d^.^22–24^ Our results, therefore, indicate that both *C*^d^_dl_ and *C*^d^_q_ contribute to the measured *C*^d^ at the chosen electrolyte concentration around Δ*ψ*^0^ = 0. This was confirmed by evaluating *C*^d^_q_ explicitly using 1/*C*^d^_q_ = 1/*C*^d^ − 1/*C*^d^_dl_, as shown in Fig. S23,† which indicates that *C*^d^_q_ is of the same magnitude as *C*^d^_dl_, on average, around Δ*ψ*^0^ = 0.

Increasing the potential difference results in rapidly increasing *C*^d^_q_, with a larger magnitude in the gradient (d*C*^d^_q_/dΔ*ψ*^0^) on the anodic branch of the *C*^d^_q_(Δ*ψ*^0^) curve. This means that, at large values of *σ*, *C*^d^_dl_ dominates the measured *C*^d^ at graphite. Indeed, the shape of *C*^d^_q_(Δ*ψ*^0^) matches well to experimental measurements of the ‘quantum capacitance’ at graphene electrodes, although the size of *C*^d^_q_ is an order of magnitude greater here at similarly large values of applied potential.^90^ This is due to the proportional increase in the electrode (electron) density of states per unit surface area that occurs as the number of graphene layers are increased, as determined in recent simulations at the DFT level.^91^ The DFT calculations show that a *C*^d^_q_ of around 10 μF cm^−2^ (at the *C*^d^_q_ minimum) in 6-layer graphene is increased by an order of magnitude when |Δ*ψ*^0^| ≈ 0.5 V, in close agreement to the evaluated *C*^d^_q_ here.

Early models used to explain the *C*^d^ at graphite implicitly assumed monotonic behaviour in the concentration profiles of ions in solution adjacent to the electrode and an absence of specific adsorption, through their use of the GCS model when apportioning contributions to *C*^d^.^21–24^ This was motivated by that fact that the minimum *C*^d^ of graphite is approximately an order of magnitude smaller than the *C*^d^ measured for metal electrodes at comparable conditions. It was, therefore, implicitly assumed that *C*^d^ was dominated by *C*^d^_q_.^21,22,24^ Outside of the GCS model, however, there is no reason to assume that graphite electrodes accumulate solution charge in the same way as metal electrodes, such that they should have similar magnitudes of *C*^d^_dl_; indeed, this was briefly considered early on.^23,24^

Our estimates of *C*^d^_dl_ are, to some degree, force field dependent. At the concentrations adopted, however, it is likely that other force fields, which capture (at least implicitly) the polarisation at the graphite surface, will determine a similar value for *C*^d^_dl_ due to the partial ‘saturation’ of the multi-layered double layer structure at high concentrations. In addition, the possible adsorption of airborne contaminants^27^ at the surface, and the effect of steps and other surface defects in HOPG should be considered when attempting any comparison between simulations and experiments. Nonetheless, our results are consistent with a dependence of *C*^d^ on the solution composition, reported here and elsewhere.^25,26^ Asymmetries in the tails of the *C*^d^ curves are accentuated in the simulation results, and these are apparent also in experiments—highlighted more clearly by the changing gradients of the *C*^d^(Δ*ψ*^0^) curve in Fig. S22B.† This is a common feature in studies of ionic liquids,^58^ where the anisotropic, bulky charge carriers saturate opposing surface charge densities differently.

## 3 Conclusions

Little is known about the effect of concentration on the structure and dynamics of electrolytes in molecular detail at carbon surfaces over a wide range of bulk concentrations and how these affect the electrochemical properties of the interface. Here, CμMD simulations were applied to simulate the graphite–NaCl(aq) interface in equilibrium with constant concentration, electroneutral bulk solutions sampling a range of electrolyte concentrations (0.2–9.2 M). Na^+^ accumulation at the graphite surface in a dynamically adsorbed first layer (as confirmed by analyses of ion diffusion coefficients) results in an effective positive surface charge density that is charge-compensated by the accumulation of Cl^−^ ions. At the lowest bulk electrolyte concentrations, the concentration of anions decreases exponentially (exponential fit *R*^2^ = 0.94 at 0.2 M), with the double layer thickness shrinking with increasing concentration. Above 0.6 M, however, a transition in the screening behaviour occurs, with alternating layers of cations and anions (up to four–five solvent layers in extent) forming before the effective surface charge is neutralised, leading to an increasing double layer size with concentration. The concentration for this transition is in good agreement with experimental studies which found a minimum in the NaCl(aq) electrostatic screening length at 0.1–0.9 M.^28^ The crowding of ions, and increasing over-screening in the double layer region, manifests in a reduction to the change in the potential of zero charge with incremental changes to the bulk electrolyte concentration, which was confirmed experimentally.

Charging the graphite surface, when *c*^b^_NaCl_ ≈ 1 M, allowed for evaluation of the double layer differential capacitance (*C*^d^). That the average over the same region of applied potential in simulations (6.1 ± 1.2 μF cm^−2^) and experiments (5.54 ± 0.60 μF cm^−2^) were of the same order of magnitude highlights a problem with previous analyses of the double layer capacitance (*C*^d^_dl_) of the graphite–electrolyte interface, which assumed that a GCS model was applicable.^22,24^ We emphasise that parallels cannot be drawn between the classical picture of the compact layer capacitance at metal electrodes and the graphite electrode; this led us to reconsider the relative contributions to the total *C*^d^. Estimates of *C*^d^ associated with the response of the graphite electronic structure to charging (*C*^d^_q_), using our simulated values for *C*^d^_dl_ and total measured *C*^d^, shows that *C*^d^_q_ is of a similar magnitude to *C*^d^_dl_ around Δ*ψ*^0^ = 0 (around 10 μF cm^−2^), increasing substantially with the value of applied potential. This is in line with recent simulations of many-layer graphene.^91^ The significance of this result is that *C*^d^ (at moderate-to-high electrolyte concentrations) does not simply report on the density of states of the graphite material, as has become the accepted norm in much of the electrochemistry literature.

Evaluation of interfacial molalities showed increases of up to six times the levels of the bulk solution in regions confined to the double layer, where the solution mass densities were up to three times the levels of the bulk (1030 kg m^−3^ at 1 M). Because both cations and anions adsorb in the double layer, a complex solution structure emerges which affects the thermodynamic and aforementioned electrochemical properties of the interface. Despite the high molalities, the mean ion activities are reduced due to the large local electric fields present, emphasising the non-ideality of this region. Cluster analyses reveal the presence of enhanced clustering at concentrations above 5 M, which has implications for the rates of surface-driven reactions. The clusters are reminiscent of those identified in simulations at the limit of solution stability in bulk solutions (∼15 mol kg^−1^), where phase separation becomes spontaneous.^83^ Given that the clusters emerge in the metastable solution (*i.e.*, beyond the nominal solubility of NaCl in the bulk and below the limit of solution stability), further investigation is required to determine their role in salt precipitation facilitated by surfaces.

The insight gained here for planar carbon interfaces also applies to the wide range of activated carbon materials, which have graphitic microdomains. Further work will be required to understand how the structure at the planar interface is modified by the complex, confined geometry and chemical heterogeneity of such materials.

## Data availability

GROMACS and Plumed Input and example output files, including the force field parameters necessary to reproduce the simulation results reported in this paper, are available on github (https://github.com/aaronrfinney/CmuMD-NaCl_at_graphite). The PLUMED input files are also accessible *via* PLUMED-NEST (https://www.plumed-nest.org^92^), the public repository for the PLUMED consortium, using the project ID: plumID:21.011. Details on how to use and implement the CμMD method within PLUMED is available on github (see https://github.com/mme-ucl/CmuMD).

## Author contributions

All authors designed the research and wrote the manuscript. A. R. F. and I. J. M. performed the research and analyses. P. R. U. and M. S. coordinated the research.

## Conflicts of interest

There are no conflicts to declare.

## Supplementary Material

SC-012-D1SC02289J-s001
