# Supplementary material for: Electrochemistry, ion adsorption and dynamics in the double layer: a study of NaCl(aq) on graphite
Source: Chem Sci. 2021 Jul 14;12(33):11166–80. doi: 10.1039/d1sc02289j (PMC8386640; doi:10.1039/d1sc02289j)
Supplement: SC-012-D1SC02289J-s001 [file SC-012-D1SC02289J-s001.pdf]

Electronic Supplementary Material (ESI) for Chemical Science.
This journal is © The Royal Society of Chemistry 2021

Electrochemistry, Ion Adsorption and Dynamics in the Double Layer: A Study of NaCl(aq) on Graphite

Supporting Information

Aaron R. Finney[†,*], Ian J. McPherson,[‡] Patrick R. Unwin,[‡] and Matteo Salvalaglio[†,*]

[†] *Thomas Young Centre and Department of Chemical Engineering, University College London, London WC1E 7JE, United Kingdom*

[‡] *Department of Chemistry, University of Warwick, Coventry, CV4 7AL, United Kingdom*

[*] To whom correspondence may be addressed: a.finney@ucl.ac.uk; m.salvalaglio@ucl.ac.uk

Contents

A Materials and Methods **2**
A.1 Simulation Details . . . 2
Simulation setup. . . . 2
CμMD. . . . 2
Force field. . . . 2
Applied surface charge. . . . 3
Structural analyses using Plumed. . . . 4
Diffusion coefficients. . . . 4
Additional files. . . . 4
A.2 Experimental Details . . . 4

B Theoretical Background **5**

C CμMD Bulk Concentrations **7**

D Double Layer Size from Solution Mass Densities **7**

E Models for the Solution Permittivity Orthogonal to Graphite **8**

F Additional Figures **10**

References **24**

A Materials and Methods

A.1 Simulation Details

Simulation setup. A 5.4×5.5×2.7 nm graphite supercell was created from the unit cell structure elucidated by Trucano and Chen,[1] after first transforming the unit cell to an orthorhombic geometry. The resulting structure contained eight layers of graphene positioned perpendicular to the simulation cell x axis. Two different systems were prepared, namely system A and B. In system A, 11,389 water molecules, 418 Na^+ and 418 Cl^- ions were added to the simulation cell above and below the graphite with respect to x. With carbon atoms restrained to their crystalline lattice positions, a molecular dynamics (MD) simulation was performed for 0.2 ns at 298 K and 1 bar to relax the solution and equilibrate the simulation cell volume. The resulting solution mass density was 1.05 g cm^{-3} with an ion molality, b(NaCl) = 2 mol kg^{-1}. System B was prepared following the same protocol using 1,672 NaCl and 13,819 water molecules, leading to a solution mass density of 1.19 g cm^{-3} and ion molality b(NaCl) = 6.7 mol kg^{-1}.

MD simulations were performed using the leapfrog time integration algorithm with a 2 fs timestep in the GROMACS 2018.6 package [2]. The temperature and pressure of the system were held constant within statistical fluctuations using the Bussi-Donadio-Parrinello thermostat [3] and the barostat of Berendsen et al. [4] The internal degrees of freedom of water molecules were constrained using the LINCS algorithm [5]. Smooth particle mesh Ewald summation [6] was adopted to compute atomic energies and forces arising from electrostatic interactions, where the real-space contributions were computed for atoms within 0.9 nm. Lennard-Jones interactions were truncated at 0.9 nm with a dispersion correction added to the energies of short-range intermolecular interactions. Periodic boundaries were imposed in three-dimensional Euclidean space.

To create the initial configurations for CμMD simulations [7]—where an internal, high salinity region far from the graphite surface is used as a reservoir to control the concentration in the system—the distances between all ions and graphite carbon atoms were restrained to 6 nm using a harmonic bias potential with a force constant of 3×10^5 kJ mol^{-1}. Systems A and B were simulated for 0.5 ns in the canonical ensemble under the effect of this restraint, imposed using the PLUMED plugin (version 2.5) [8].

CμMD. CμMD [7] was adopted to maintain a constant number density of ions, n^t, in the control region (CR) solutions, relatively far from the graphite/solution interface (see Figure S1), in simulations where the total volume was held constant. The number density is controlled by applying a continuous, external force, F_i^μ, to ions at x_F: the boundary between the CR and the reservoir. When the simulation is initiated, ions from the reservoir diffuse towards the interface and F^μ acts as a semi-permeable membrane to maintain a constant, predefined value of cation and anion number densities in the CR:

$$F_i^\mu(x) = k_i(n_i^{\mathrm{CR}} - n_i^t)G(x) \tag{1}$$

Here, n_i^{CR} is the ion i number density in the CR. A continuous force is applied to ions at a fixed distance, x_F, from the midpoint (x_0) of the line spanning the x axis:

$$G(x) = \frac{1}{4\omega}\left[1 + \cosh\left(\frac{x - x_F}{\omega}\right)\right]^{-1} \tag{2}$$

In these simulations, the graphite–solution interface does not change its position in time i.e., the position x_F is time-independent. Note that an alternative approach, where the position at which the force is applied evolves during the simulation, has been adopted in CμMD studies in which a phase transition takes place at the solid/liquid interface which evolves in time[7]. In Equation 2, ω controls the width of the force region acting on particles close to x_F. In the CμMD simulations here, ω was 0.01% of the total size of x and $k = 2\times10^5$ kJ mol^{-1}; hence, the force is localised to a very narrow region in x. The size of the CR in x was 2.2 nm and $x_F = x_0 \pm 3.7$ nm.

CμMD simulations were performed for 100 ns setting n^t to 0.06022–0.6022 nm^{-3} in 0.06022 nm^{-3} increments in system A and n^t = 0.6022–6.022 in 0.6022 nm^{-3} in system B. The densities correspond to target ion molar concentrations, $c^t = n^t \times 10^{24}/N_{\mathrm{A}}$ (where N_{A} is Avogadro's constant), of 0.1–10 M (mol dm^{-3}). Typically, 20–35 ns of simulation time was required to reach a steady state in ion concentration profiles; we therefore utilised the final 50 ns of simulation trajectories in any analyses.

Force field. NaCl(aq) was simulated using the Joung and Cheatham force field [9] which adopts the extended simple pint charge (SPC/E) model [10] for water. This model reproduces reasonably well the thermodynamics of NaCl in water.[11] Graphite C–C atomic interactions were modelled using the OPLS/AA force field [12]. C–water intermolecular interactions were modelled using the atom pair potential provided by Wu and Aluru [13] based on fitting to water adsorption energies from random phase approximation calculations[14]. This model predicts a water contact angle of 42°, which the authors highlight compares favourably to experimental measurements[15, 16]. Other experiments[17] suggest a less hydrophilic wetting angle of 64° on freshly cleaved

HOPG that increases over time due to contamination in the atmosphere. The model reportedly predicts well the radial breathing mode frequency of single walled carbon nanotubes. [13] Finally, (graphite) C–Na^+ and C–Cl^- intermolecular interactions were modelled using the atom pair potentials from Williams et al.[18]. These potentials were fitted to the energies of interaction of ions with aromatic, planar molecules containing 54 carbon atoms and with solvent implicitly modelled using a conductor-like polarisable continuum. Importantly, there is a self-consistency associated with different components of the force field; for example, the ion–water model adopted in the derivation of C–ion potentials was the Joung and Cheatham force field and SPC/E water was used in the fitting of C–water interactions.

Applied surface charge. Simulations to explore graphite surface charge effects were performed where, unless otherwise stated, the simulation input parameters were the same as for CμMD simulations in the absence of a graphite surface charge. In total, 24, 100 ns simulations were initiated from a CμMD simulation in the absence of a graphite surface charge and with $c^{\mathrm{b}}_{\mathrm{NaCl}} = 1.05 \pm 0.03$ M. Surface charges were applied uniformly to the outermost carbon graphite plane. The sign of the charge was opposite on different sides of the basal planes of the graphite slab; hence, surfaces with a negative and positive surface charge density, σ, were exposed to NaCl(aq). Any induced dipole moment in x from the additional surface charges can be screened by the redistribution of charges within the internal reservoir. This approach to exploring electrified interfaces in simulations is somewhat simplistic; nonetheless, it allows for a direct appraisal of the GCS model. More sophisticated simulation methods are available to e.g., control the electric potential [19] or to capture any dynamic electronic effects at the interface [20, 21]; however, these are costly and do not ensure a constant concentration of cations and anions in the bulk.

The basal plane surface area was 29.75583 nm^2 and absolute values of charge $|q| = 0.0001$–$0.1e$ were applied to the 1144 outermost carbon atoms. This resulted in absolute surface charges densities, $|\sigma| = 0.0038$–$3.8e$ nm^2. However, beyond $|\sigma| =1$ e nm^2, over-depletion of ions in the reservoir region occurred (see Figure S16). We therefore limited our analyses to systems where $|\sigma|$ was below $1e$ nm^2. Table S1 provides all of the surface charge densities analysed. Electric fields from the applied surface charges were determined using the Poisson Equation (Equation 6 in Section B). Using a constant relative permittivity, $\varepsilon_r = 71$, to model the solvent medium, Grahame's Equation (Equation 13) predicts a potential drop across the interface, $\psi^0_{\mathrm{Grahame}}$, of 0.28–58 mV. As discussed in the main text, Grahame's Equation makes inaccurate assumptions about the double layer structure at intermediate–high concentrations; the potential change, therefore, is underestimated using this approach.

$\|q\|$ (e atom^{-1})	$\|\sigma\|$ (e nm^2)	E (V nm^{-1})
0.0001	0.0038	0.07
0.0002	0.0077	0.14
0.0005	0.0192	0.35
0.0008	0.0308	0.56
0.0010	0.0384	0.70
0.0015	0.0577	1.04
0.0020	0.0769	1.39
0.0040	0.1538	2.78
0.0060	0.2307	4.17
0.0080	0.3076	5.57
0.0100	0.3845	6.96
0.0120	0.4614	8.35
0.0140	0.5382	9.74
0.0160	0.6151	11.13
0.0180	0.6920	12.52
0.0200	0.7689	13.91
0.0220	0.8458	15.31
0.0240	0.9227	16.70
0.0260	0.9996	18.09

Table S1: Applied surface charges, q, and surface charge densities, σ, used in simulations in this work. Electric fields, E, calculated using the Poisson Equation (where $\varepsilon_r = 1$) are also provided.

Structural analyses using Plumed. Average first-sphere coordination numbers, N_{i-j}, between atoms i and j were calculated using a continuous but sharp definition of coordination implemented in Plumed: [8]

$$N_{i-j} = \frac{1}{M_i} \sum_{i=1}^{M_i} \sum_{j=1}^{M_j} \frac{1 - \left(\frac{r_{ij}}{r_0}\right)^{32}}{1 - \left(\frac{r_{ij}}{r_0}\right)^{64}} \tag{3}$$

where M_i and M_j are the numbers of atoms of types i and j, respectively; r_{ij} are the distances between ion pairs i and j; and, r_0 were set to 0.355, 0.325 and 0.385 nm for the pairs Na–Cl, Na–Ow and Cl–Ow (where Ow is oxygen of water), respectively. Figure S13 highlights that the chosen definitions of coordination ensured that only atoms in direct contact were considered when calculating the mean coordination numbers.

To analyse ion clusters, we adopted a depth first search algorithm [22] to identify clusters from adjacency matrices built using the above definition of first-sphere coordination between ions. Clusters were defined using a continuous switching function to identify ion associates containing two or more ions. Instructions to access the Plumed input files for both coordination and cluster analyses are provided at the end of this section.

Diffusion coefficients. The diffusion coefficients, D, for ions and water were calculated using the Einstein relation: $D = \lim_{t\to\infty} d < (\mathbf{r}(t) - \mathbf{r}(0))^2 > /6dt$, where $\mathbf{r}(t)$ is the position of a particle at time t. As a reference, D for ions and water were calculated from a simulation of bulk NaCl(aq) at 1 M. In a simulation of NaCl(aq) containing 148 ions and 4000 water molecules in a cubic cell simulated for 10 ns at 298 K and 1 atm, $D = 0.94 \pm 0.07, 1.26 \pm 0.08$ and $2.17 \pm 0.03 \ \times 10^{-5}$ cm^2 s^{-1} for Na^+, Cl^- and O of water, respectively. When the correction of Yeh and Hummer is applied to account for the finite size effects of simulationn cells,[23] (using the shear viscosity of SPC/E water from Reference [24]) the values of the corrected diffusion coefficients were $1.11, 1.44$ and $2.35 \ \times 10^{-5}$ cm^2 s^{-1}. This makes the calculated mean ion diffusion coefficients slightly smaller than those calculated by Joung and Cheatham but within the uncertainty estimates. [25]

Diffusion coefficients for ions and water were measured in $\Delta x = 0.4$ nm windows moving away from the graphite surface in 50×1 ns calculations, with D calculated by fitting to data when $t = 100 - 600$ ps. This procedure was followed due to the propensity for ions and water molecules to translate to adjacent windows in x on the timescales of the simulations. Mean D were therefore estimated by averaging many shorter trajectories where the position of atoms at $t = 0$ determined their contribution to mean D values. We also calculated the x component of the diffusion coefficients (D_x) to determine the mobility of molecular species when the electrical properties of the solution are changing in the double layer region.

Additional files. GROMACS and Plumed input and example output files, including the force field parameters necessary to reproduce the simulation results reported in this paper, are available on github (see https://github.com/aaronrfinney/CmuMD-NaCl_at_graphite). The PLUMED input files are also accessible via PLUMED-NEST (www.plumed-nest.org [26]), the public repository for the PLUMED consortium, using the project ID, plumID:21.011. Details on how to use and implement the CμMD method within PLUMED is available on github (see https://github.com/mme-ucl/CmuMD).

A.2 Experimental Details

The differential capacitance, C^d, of the NaCl(aq)–graphite system was measured via electrochemical impedance spectroscopy (EIS) using a droplet configuration. The top face of an HOPG crystal (1 cm^2, SPI-1 grade, Mosaic spread angle $0.4° \pm 0.1°$; Structure Probe, Inc.) was exfoliated by peeling off a layer attached to adhesive tape (Scotch Tape, 3M). A 6 mm inner diameter silicone O-ring was placed on the freshly exfoliated face into which was pipetted a 200 µL droplet of NaCl(aq) (99.999% metal basis, Sigma Aldrich) solution (18.2 MΩ cm, Purelab Chorus, Elga). A reference electrode (leak-free Ag/AgCl, 3.4 M KCl, Innovative Instruments Ltd.) and a counter electrode (0.5 mm diameter Pt wire) were then quickly lowered into the droplet and the EIS was commenced. As mentioned in the main text, the freshly exfoliated graphite surface is highly susceptible to adsorption of organic impurities and degrades rapidly with time.[27, 28] The impedance was therefore measured at a single frequency (105 Hz) using a potentiostat (compactstat, Ivium Technologies B.V.) with a 10 mV perturbation to minimise measurement time. Preliminary experiments showed that the impedance at this frequency was dominated by capacitance (the phase angle, $\phi \approx -89°$) and was relatively insensitive to the frequency used over the concentration range studied. ϕ remained approximately constant throughout the potential sweep. C^d was therefore calculated directly from the imaginary part of the complex impedance, Z", using $C^d = -(2\pi Z" f A)^{-1}$, where f is the frequency of the perturbation and A is the geometric area defined by the O ring. [29] Small deviations in ϕ between sweeps did not lead to significant changes to C^d, given the existing error bars. The values of C^d measured agree well with those of previous measurements in 0.5 M NaCl.[25]

Two protocols were adopted to measure the capacitance. Protocol A started from -0.5 V vs Ag/AgCl, with 5 s equilibration at this potential before the impedance was measured. The potential was then increased by 10

mV and the process repeated until the potential reached 0.0 V vs Ag/AgCl. In this way, the 51 potentials could be measured in several minutes, minimising the time that the HOPG was exposed to solution and, therefore, limiting the effect of potential organic impurity adsorption. Preliminary experiments, in which C^d was measured as the potential was first incremented and then decremented, already showed a slight hysteresis indicative of surface deactivation. Longer delays between measurements of 20 minutes showed substantial changes to both the magnitude and shape of the C^d–potential curve, in agreement with previous work,[31] and confirming the need to proceed with measurements quickly. Protocol B aimed to measure over a wider potential window and replicate the more common potential resolution used for measuring C^d–potential curves with 50 mV increments to the applied potential, initiated at -0.75 V vs Ag/AgCl. Protocol A was repeated at each sampled concentration, allowing uncertainties in C^d (Figure S2) to be estimated from the standard deviation of the measured data in three repeat experiments. Any curves that deviated from the common double minimum shape also showed much lower capacitance values and were therefore rejected.

Due to variations in the exposed surfaces formed during each exfoliation, the absolute magnitude of C^d varies by 1–2 µF cm^{-2} between measurements, as was seen previously,[31] with the value of C^d at the minimum being the same at all concentrations (within error). The more negative of the two minima in C^d was extracted for each repeat and used to calculate the mean and standard deviation of the C^d minimum at each concentration. While it has been shown that asymmetric ion adsorption causes the PZC to shift away from the C^d minimum, both change in the same direction and by similar amounts, and converge at high concentration, allowing min(C^d) to function as an effective proxy for the PZC in our work.[32] The shift in the C^d minimum can also be seen in the data collected using Protocol B (see Figure S3), although the double minimum feature observed with Protocol A is not visible at this sampling resolution.

B Theoretical Background

Models to describe the double layer began with the work of Helmholtz in the 1870's.[33] Since then, focus has largely been devoted to the effect of charged electrodes in contact with electrolyte solutions. Helmholtz suggested that a static, compensating layer of ions from the extended liquid phase adsorbs at a surface to neutralise the total surface charge. This so-called electrical condenser predicts a linear change in the electric potential, ψ, across a distance determined by the radius of the adsorbed charge carriers. The Helmholtz model fails to account for the thermal motion of ions at the interface.

Gouy and Chapman provided the earliest theory able to account for the entropy of charge carriers in solution adjacent to a charged planar surface.[34, 35] Gouy-Chapman theory predicts that a diffuse ion layer assembles at the interface with a solid substrate. The surface charge density, σ, is thus compensated by the total charge density, ρ, in the liquid phase in the direction, x, orthogonal to the surface:

$$\sigma = -\int_0^\infty \rho(x)\, dx \tag{4}$$

Ions in the diffuse layer dissipate the electric potential of the double layer by adopting a structure normal to the surface consistent with a Boltzmann distribution:

$$n_i(x) = n_i^{\mathrm{b}} \exp\left(-\beta z_i e \psi(x)\right) \tag{5}$$

where n_i is the ion i number density and n_i^{b} is the uniform ion number density in the extended liquid phase (i.e. the bulk of the solution); $z_i e = q_i$, which is the electric charge of i with z_i valency and e is the elementary unit of charge; and, $\beta = 1/k_{\mathrm{B}}T$ where k_{B} is Boltzmann's constant and T is temperature. The above equation highlights that the work required to bring ion i from the extended liquid phase to the interfacial region, $W_i(x) = -z_i e\psi(x)$; hence, a fundamental assumption is that only electrical work is involved. [36]

The dependence of ion density distributions on ion charges implies a local departure from electroneutrality within the diffuse layer. The resulting ψ and electric field, E, in the double layer can be calculated using the Poisson equation from the charge densities and permittivity of the medium, $\varepsilon = \varepsilon_0 \varepsilon_r$ (where ε_0 and ε_r refer to the permittivity of a vacuum and the relative permittivity of the solution medium, respectively):

$$\frac{d^2\psi(x)}{dx^2} = \frac{d\mathrm{E}(x)}{dx} = \frac{-\rho(x)}{\varepsilon} \tag{6}$$

noting that $\rho = \sum_i z_i e n_i$. Combining equations 5 and 6 leads to the non-linear Poisson-Boltzmann equation (PBE) for a binary univalent electrolyte ($|z_{\mathrm{cation}}| = |z_{\mathrm{anion}}| = 1$ and $n^{\mathrm{b}} = n^{\mathrm{b}}(\mathrm{cation}) = n^{\mathrm{b}}(\mathrm{anion})$):

$$\frac{d^2\psi(x)}{dx^2} = \frac{en^{\mathrm{b}}}{\varepsilon}\left[\exp\left(\beta e\psi(x)\right) - \exp\left(-\beta e\psi(x)\right)\right] \tag{7}$$

This self-consistent, second order differential equation directly relates the charge distribution of ions to the potential of mean force associated with bringing a point charged ion into the diffuse layer (due to Coulombic

intermolecular interactions). In typical applications of the PBE, mean field theory applies: local ion–ion and ion–solvent correlations are neglected and the electric potential is due to the thermally averaged electric field. Indeed, in this description, the solvent is implicit and is often modelled assuming a fixed value of ε. At the inner- and outer-most boundaries of the double layer, the following conditions apply, $\mathrm{E}^0 = -\sigma\varepsilon^{-1}$ and $\mathrm{E}^\infty = 0$.

To obtain the electrolyte densities at the electrode surface (n_i^{s}), the derivative of Equation 5 with respect to x can be written as,

$$\frac{dn_i}{dx} = \frac{\varepsilon\beta}{2}\frac{d}{dx}\left(\frac{d\psi(x)}{dx}\right)^2 \tag{8}$$

by adopting the relationship in Equation 7 for a generic ion type. [36] Integrating Equation 8 from the extended liquid phase to any x position in the diffuse layer results in,

$$\sum_i n_i(x) = \sum_i n_i^{\mathrm{b}} + \frac{\varepsilon\beta}{2}\left(\frac{d\psi(x)}{dx}\right)_x^2 \tag{9}$$

Making use of the relationships in Equations 6 and 4, Equation 9 can be rewritten as,

$$\sum_i n_i^{\mathrm{s}} = \sum_i n_i^{\mathrm{b}} + \frac{\sigma^2\beta}{2\varepsilon} \tag{10}$$

Equation 5, and Equation 7 by association, indicate a diffuse layer of infinite size: $c_{\mathrm{cation}} = c_{\mathrm{anion}}$ when $\psi(x \to \infty) = 0$. Yet, the extent to which n_i decay and converge to n^{b} within thermal fluctuations can be approximately determined. The size of the diffuse layer can be calculated according to the Gouy length, λ_{G}, which is determined as a solution to Equation 7:

$$\lambda_{\mathrm{G}} = \frac{2\kappa^{-2}}{\exp(\beta e\psi(x)/2) + \exp(-\beta e\psi(x)/2)} \tag{11}$$

Here, κ^{-1} is the Debye length which takes the functional form,

$$\kappa^{-1} = \left(\frac{\varepsilon}{2\beta e^2 n^{\mathrm{b}}}\right)^{\frac{1}{2}} \tag{12}$$

The Gouy length decreases exponentially as σ is increased; however, at the highest surface charge densities, this can be approximated as $2\varepsilon/\beta e\sigma$. [37] As σ approaches zero, λ_{G} and κ^{-1} converge (at constant n^{b}). Indeed, for surfaces with a low applied potential—where $e|\psi| < k_{\mathrm{B}}T$—the Debye length is assumed to be the characteristic decay length over which ion concentrations converge to n^{b}. The size of the double layer, therefore, decreases as n^{b} increases. When dealing with moderate electrolyte concentrations or surface charge densities, these simple models offer limited predictive power. A general solution to the Poisson-Boltzmann equation can be provided for ideal solutions at perfectly planar, homogeneously charged surfaces.[38] The added complexity of non-ideal electrolyte solutions and imperfect surface geometries makes predicting the size of the diffuse layer a considerable challenge.

To relate the surface charge density to the surface potential, one can employ Equation 4 with Equation 7 rewritten using a hyperbolic trigonometric function, leading to a notable result known as Grahame's equation[39]:

$$\begin{aligned}\sigma &= -\int_0^\infty \rho(x)\,dx = -\varepsilon\left.\frac{d\psi(x)}{dx}\right|_{x=0} \\ &= 2\varepsilon\kappa\sinh\left(\frac{\beta e\psi(0)}{2}\right) \\ &= \sqrt{8\varepsilon n^{\mathrm{b}}\beta^{-1}}\sinh\left(\frac{\beta e\psi^0}{2}\right)\end{aligned} \tag{13}$$

The Stern modification to the Gouy-Chapman model accounts for the finite size of ions in the immediate vicinity of the surface. In the Gouy-Chapman-Stern (GCS) model, a layer of ions adsorbed at the substrate/solution interface is introduced as a fixed plate capacitor.[40] The ions in such layers are generally considered to be immobile. Following this approach, the double layer can be divided into two regions: an innermost region called the Stern layer, where the potential decays linearly, followed by a diffuse layer, where the potential decays exponentially according to the PBE. The potential at the transition point between these two regions is associated with the ζ-potential. Given an assumed discontinuity in the mobility of charge carriers at this point, the characteristic distance from the electrode interface is described as the slipping plane. The depth of the Stern layer

is of the size of ion radii, with or without their solvation sphere(s), labelled as the inner- and outer-Helmholtz planes, respectively.

Despite their practical relevance, the simple mean-field models so far described fail to account for the asymmetric adsorption of cations and anions (with equal but opposite applied surface potential), and the role of solvent molecules—of particular importance in the case of highly polar solvents—when determining the electric potential. Moreover, it follows from Equation 10 that, in the absence of surface charges, the net total ion concentration at the interface is unperturbed compared to that in the extended liquid phase. This is inconsistent with the fact that uncharged surfaces can, and do, induce perturbations to the structure and composition of the liquid due to van der Waals forces and steric effects leading to an effective surface charge.[32, 41] The potential of zero charge, $\psi^{\rm pzc}$, encapsulates these phenomena and can be calculated using Equation 6 with zero applied surface potential. In systems containing metallic electrodes, a linear correlation in $\psi^{\rm pzc}$ with respect to the work function of the electrode is often observed.[42] According to Gouy-Chapman theory, $C_{\rm d}$ has a minimum at $\psi^{\rm pzc}$; however, specific ion adsorption leads to deviations depending upon the ionic species in contact with the electrode.[32] At high electrolyte concentrations, asymmetric capacitance–potential curves accompany a shift in the minimum capacitance, when cation and anion surface-adsorption energies differ.[32] At relatively high ionic strengths, electrolytes in the double layer can also influence the charge distribution of the substrate. This polarisation affects the value of $C_{\rm d}^{\rm Stern}$.[43]

Extensions to the simplest mean field models and alternative models to predict the electrical properties of interfaces have been proposed.[32, 44–46] Particularly relevant in this regard is the case of ionic liquids at electrified interfaces, where the anisotropy and asymmetry in the adsorption of charge carriers in the liquid phase cannot be ignored. Models which e.g., involve minimising the free energy function describing the system constructed using a Poisson–Boltzmann lattice-gas model, have been developed to describe these systems.[44, 47]. It was noted that these models are not so valuable to understand conventional electrolyte systems—such as the one that is the focus of this study—which should be modelled reasonably well by Gouy-Chapman theory.[47]

C CμMD Bulk Concentrations

Some discrepancies were observed between the targeted ion molar concentrations (c^t) and those that were measured in the control regions ($c^{\rm CR}$) of CμMD simulations following equilibration. In particular, there was a slight contraction of the targeted parameter space, with the measured $c^{\rm CR}$ being greater and smaller than c^t at the lower and higher end of the overall targeted concentration range. The deviations were most apparent when ion concentrations in the reservoir were much lower/higher than $c^{\rm CR}$ (see Table S2). Large concentration gradients between the CR and reservoir also induced fluctuations in $c(x)$ around x_F. While not important in the current work, these effects can be mitigated by varying the values of k and ω in the definition of F_i^μ, and by ensuring that the reservoir concentration does not become too low.

In all our analyses, we take $c^{\rm b}$ to be the mean NaCl concentration in a 0.5 nm stable region of the $c(x)$ profile in the CR (i.e., the mean value of $c(x)$ in the CR away from x_F). $c^{\rm b}$ is therefore the 'bulk' concentration of ions—representing the extended solution phase—in equilibrium with the solution at the graphite surface. Importantly, fluctuations in $c^{\rm CR}$ and $c^{\rm b}$ were independent of c^t, as shown in Table S2 and Figure S4. This is essential e.g., to determine the depth of the double layer region and for the accurate calculation of ion activities as a function of x.

D Double Layer Size from Solution Mass Densities

The size of the interfacial solution region can be identified by calculating the position in x where the solution mass density, $\rho_m = \sum_i m_i n_i$ (where m_i is the mass of atom i), diverges from the bulk uniform mass density. The first derivative of ρ_m with respect to x was calculated after first applying a Savitzky-Golay filter to smooth the data. The size of the interface region in solution was then determined as the distance where the fluctuations in $d\rho_m/dx$ in a 0.5 nm region moving away from the graphite surface were within $2\sigma_b$; here, σ_b is the standard deviation of $d\rho_m/dx$ in the bulk region.

The double layer size determined following the density criterion above is approximately constant (within statistical uncertainties) as a function of bulk ion concentrations. The mean value of the double layer size was 1.43 ± 0.25 nm. The result is not surprising, given that ρ_m is dominated by the density of water. The interface induces ordering of the water molecules in a relatively compact region near the graphite surface, but this does not extend beyond five molecular layers across the entire concentration range. An exception to this was the highest concentration case ($c^{\rm b}_{\rm NaCl}$ =9.8 M), where the width of the density perturbation induced by the graphite was 2.43 ± 0.47 nm. We note that at the highest bulk ion concentrations, changes to the ordering of water molecules within the double layer region emerges, as indicated by the concentration profiels in Figure S6.

	c^t (M)	$c^{\rm b}$ (M)	$c^{\rm b}/c^t$
System A	0.1	0.23(0.01)	2.3
	0.2	0.32(0.01)	1.62
	0.3	0.41(0.01)	1.38
	0.4	0.49(0.02)	1.22
	0.5	0.58(0.02)	1.17
	0.6	0.67(0.02)	1.12
	0.7	0.78(0.02)	1.11
	0.8	0.87(0.02)	1.08
	0.9	0.95(0.03)	1.06
	1.0	1.05(0.03)	1.05
System B	1.0	1.20(0.03)	1.2
	2.0	2.20(0.03)	1.1
	3.0	3.20(0.04)	1.07
	4.0	4.11(0.05)	1.03
	5.0	5.01(0.05)	1
	6.0	5.83(0.05)	0.97
	7.0	6.67(0.07)	0.95
	8.0	7.53(0.08)	0.94
	9.0	8.39(0.11)	0.93
	10.0	9.23(0.07)	0.92

Table S2: Target mean NaCl concentrations, c^t, and measured bulk NaCl concentrations, $c^{\rm b}$, in the CR regions of CμMD simulations. Uncertainties, shown in parentheses, indicate one standard deviation in the mean concentration values (taken from the final 50 ns of simulation trajectories) measured in a 0.5 nm region of the CR away from x_F.

E Models for the Solution Permittivity Orthogonal to Graphite

To evaluate the relative permittivity perpendicular to graphite in the x direction, $\varepsilon_r^\perp(x)$, it is possible to calculate,

$$\frac{1}{\varepsilon_r^\perp} = 1 - 4\pi\beta[\langle m_\perp(x)M_\perp\rangle - \langle m_\perp(x)\rangle\langle M_\perp\rangle] \tag{14}$$

where $m_\perp(x) = -\int_0^x \rho(x')dx'$ is the polarisation density and $M_\perp$ is the total dipole moment in the direction perpendicular to the surface, and the angle brackets indicate ensemble averages. [48, 49] As discussed in detail by Olivieri et al., [49] obtaining a converged $\varepsilon_r^\perp(x)$ profile using this approach, however, is non-trivial. Very long simulation times (up to 4.5 µs) are required and this approach does not account for the long range correlations in the dipole moment of water molecules. Alternative analyses involving the Kirkwood function can be used to account for these correlations, with appropriate approximations for the long range electrostatic boundary conditions used in finite sized simulations of inhomogeneous systems. [49] With this approach, Olivieri et al. observe a near-linear monotonically increasing $\varepsilon_r^\perp(x)$ from a value of around 10 in the first solution layer to the bulk value for SPC/E water of 71 approximately 4 nm from a planar graphene substrate. [49]

To model the varying $\varepsilon_r^\perp$ in solution next to graphite we fitted a polynomial function of the form $\varepsilon_r^\perp(x) = -0.4686x^3 + 0.1204x^2 + 24.656x + 1.0409$ ($R^2 = 0.999$) to the data in Ref. [49] (see their Figure 3C). The curve was aligned in x such that the value of $\varepsilon_r^\perp$ at the maximum of the first peak in water oxygen density profiles was 10. The maximum value of $\varepsilon_r^\perp$, around 4 nm from this position, is far beyond the distance in x where the screening profiles (f; see Figure 3 A in the main paper) converge to a value of zero. The $\varepsilon_r^\perp(x)$ function was used as input to Equation 3 of the main paper (see also Figure 3) to evaluate $\psi_i(x)$: the electric potential across the double layer.

It is interesting to consider the case when $\varepsilon_r^\perp = 71$ is used in Equation 3 (of the main paper), i.e., a constant permittivity for the solution medium equal to that of SPC/E water in the bulk. This is a significant approximation; nonetheless, it is one that is often adopted in models of the double layer at varying length scales in the absence of better models to describe $\varepsilon_r^\perp(x)$. A constant permittivity, furthermore, allows us to identify the causal response of the potential difference to changes associated only to the screening of ions in the double layer. Figure S10 A, in Section F below, shows the potential of zero charge ($\psi_i^{\rm PZC}$) as a function of ion bulk concentration ($c^{\rm b}$). The general shape of the curve from simulations using this approach matches well to that presented in Figure 3 C of the main paper. The negative gradient in $\psi_i^{\rm PZC}(c^{\rm b})$ when $c^{\rm b} < 3$ M is -0.011 ± 0.001 V/M; this is in reasonable agreement with the experimental data also provided in Figure S10 A.

As discussed in the main text, the change in the electric potential across the double layer can be calculated using the Poisson equation (see Equation 6 in SI Section B) when all solution charges are included in the

evaluation of $\rho(x)$. Here, we label this quantity $\Delta\psi$ to avoid ambiguity with ψ_i^{PZC}. In this case, and assuming a value of $\varepsilon_r^{\perp} = 1$, we provide $\Delta\psi$ as a function of c^{b} in Figure S10 B. This shows that $\Delta\psi(c^{\mathrm{b}})$ is approximately constant (0.4198 ± 0.004 V). A small positive gradient can be identified across the entire concentration range; however, any trends are very sensitive to fluctuations in the water density profiles. In fact, changes to the partial charges of water atoms of the order of $0.001e$ can result in changes to the observed trend. This is not surprising, given the observations, already highlighted above, surrounding convergence in the solvent charge densities that were observed elsewhere. [49]

F Additional Figures

Res. *CR* *CR* *Res.*

x_F x_F

Figure S1: An example configuration (projected along x and z Cartesian coordinates) taken from a CμMD simulation with target 0.06 nm^{-3} Na^{+} and Cl^{-} number densities in the control region, CR. Sodium and chloride ions are shown as blue and cyan spheres, respectively. Carbon atoms of graphite are shown as grey spheres. Water molecules are omitted for clarity. Red lines mark the boundaries for the CR and reservoir (Res.) regions.

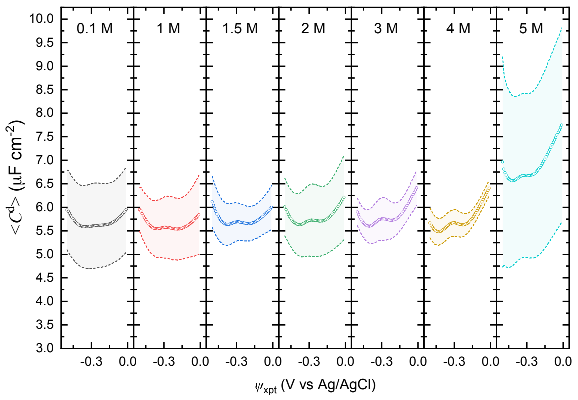

Figure S2: Experimental C^{d}-ψ_{xpt} curves for HOPG in NaCl(aq) solutions of various concentration (indicated in the figure panels) measured using Protocol A (see SI Section A). Plots show the mean (points) and standard deviation (dashed lines) of 3 measurements (or 2 measurements at 1 M and 3 M).

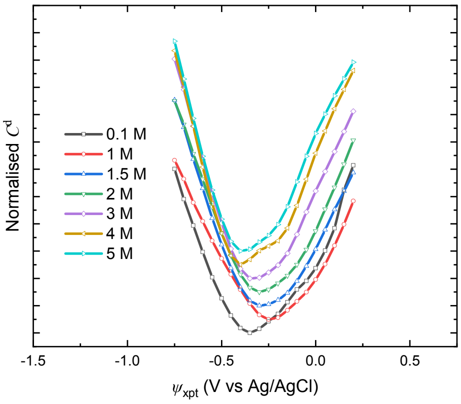

Figure S3: Experimental C^d-ψ_{xpt} curves for HOPG in NaCl(aq) solutions of various concentration (indicated in the plot) measured using Protocol B (see SI Section A). For clarity C^d has been normalised to the minimum value and the curves offset to show how the minimum shifts with changing bulk electrolyte concentrations.

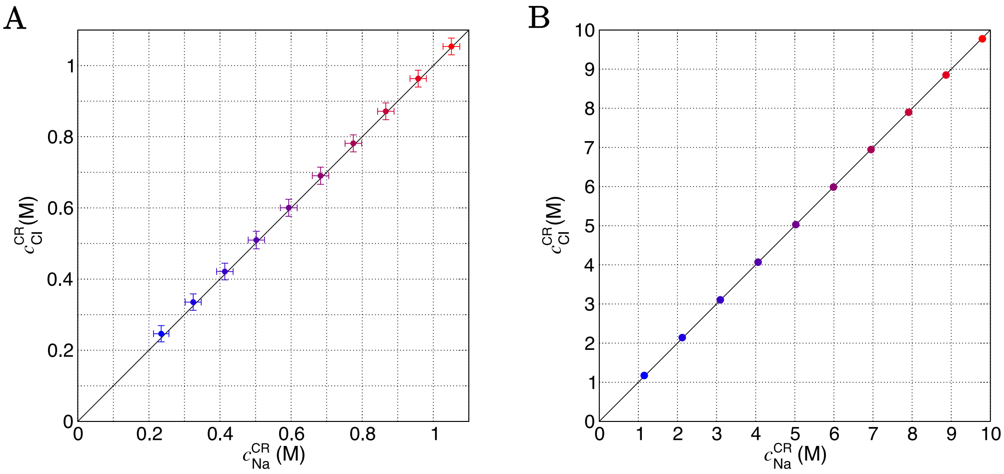

Figure S4: Na^+ and Cl^- ion concentrations measured in the control regions of CμMD simulations (c^{CR}). A provides data from simulations where target concentrations, c^t = 0.1–1 M in 0.1 M increments. B provides data from simulations where c^t = 1–10 M in 1 M increments. The concentration range is highlighted by the blue→ red colour scale indicating increasing c^t. Error bars (smaller than the data points in B) highlight uncertainties of one standard deviation in the data gathered from the final 50 ns of simulation.

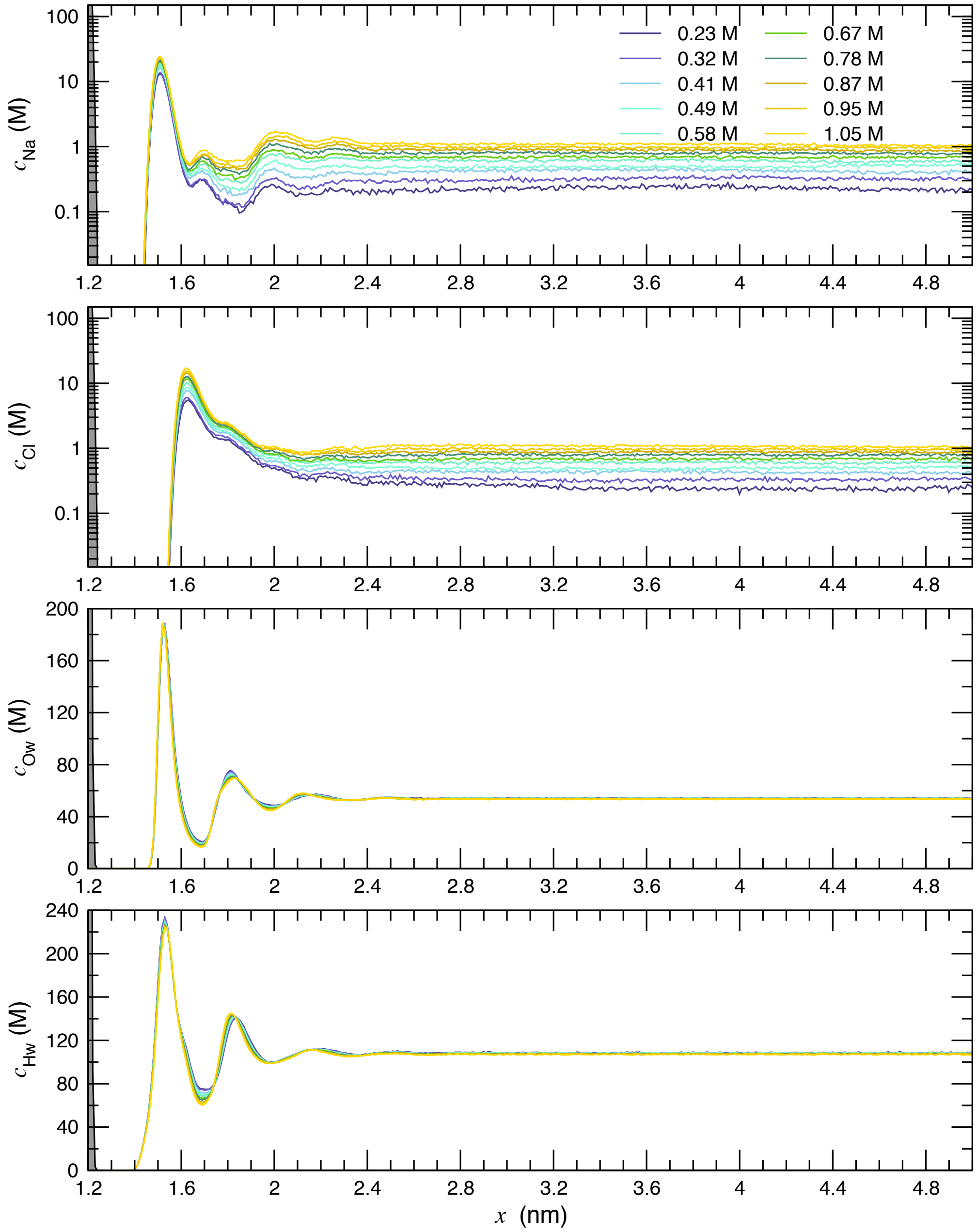

Figure S5: Averaged atom molar concentrations, c, measured for Na^+, Cl^-, Ow (water oxygen) and Hw (water hydrogen) atoms in CμMD simulations of System A (the lower end of the total concentration range; see SI Section A) as a function of distance, x, from the midpoint in the simulation cell. The colour scale indicates the mean molarity of ions in the bulk region as indicated by the key in the top panel. Ion concentrations are provided using a logarithmic scale. The edge of the carbon surface is highlighted by the grey peak on the left of the x axis.

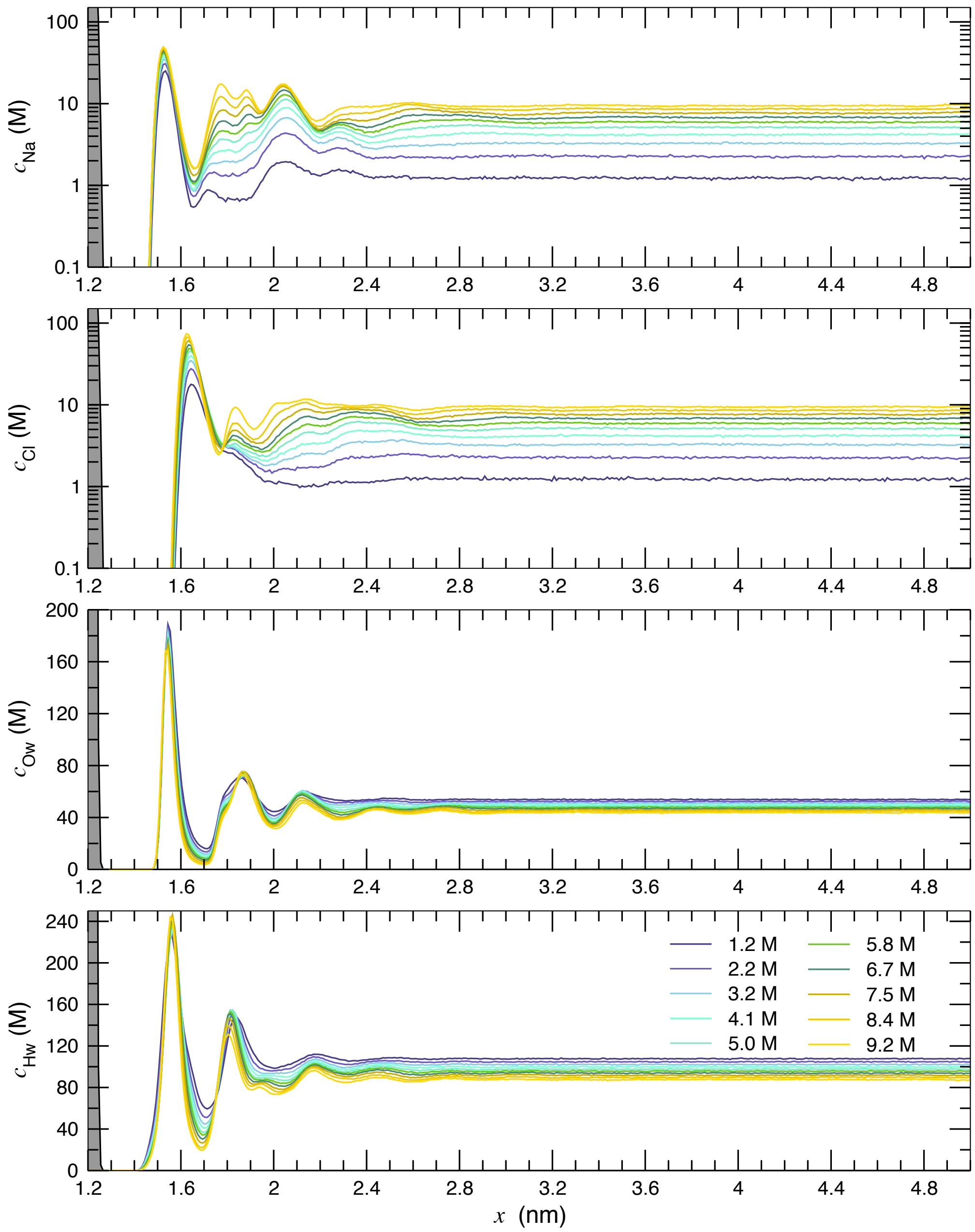

Figure S6: Averaged atom molar concentrations, c, measured for Na^+, Cl^-, Ow (water oxygen) and Hw (water hydrogen) atoms in CμMD simulations of System B (the upper end of the total concentration range; see SI Section A) as a function of distance, x, from the midpoint in the simulation cell. The colour scale indicates the mean molarity of ions in the bulk region as indicated by the key in the bottom panel. Ion concentrations are provided using a logarithmic scale. The edge of the carbon surface is highlighted by the grey peak on the left of the x axis.

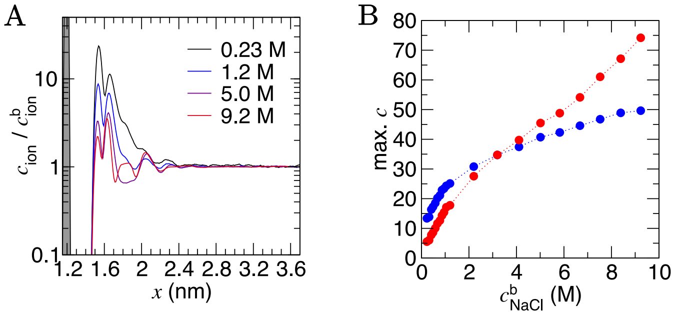

Figure S7: A: Total ion concentrations ($c_{\rm ion} = c_{\rm Na} + c_{\rm Cl}$) as a function of x, normalised by concentrations in the bulk ($c^{\rm b}_{\rm ion}$) as indicated by the key. B: Maximum Na^{+} (blue) and Cl^{-} (red) concentrations in the interfacial region taken from the concentration profiles in Figures S5 and S6

.

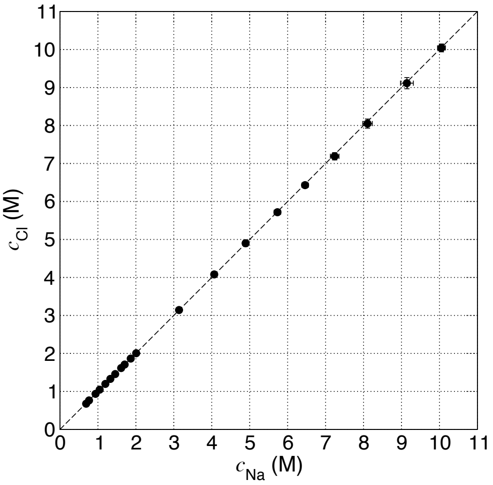

Figure S8: Na^{+} and Cl^{-} ion concentrations measured in the interface regions of CμMD simulations ($c^{\rm i}$). The interface was identified as a 1.5 nm region from the distance of closest approach of ions to the graphite surface in the solution phase. Error bars indicate uncertainties of one standard deviation in the data gathered from the final 50 ns of simulation.

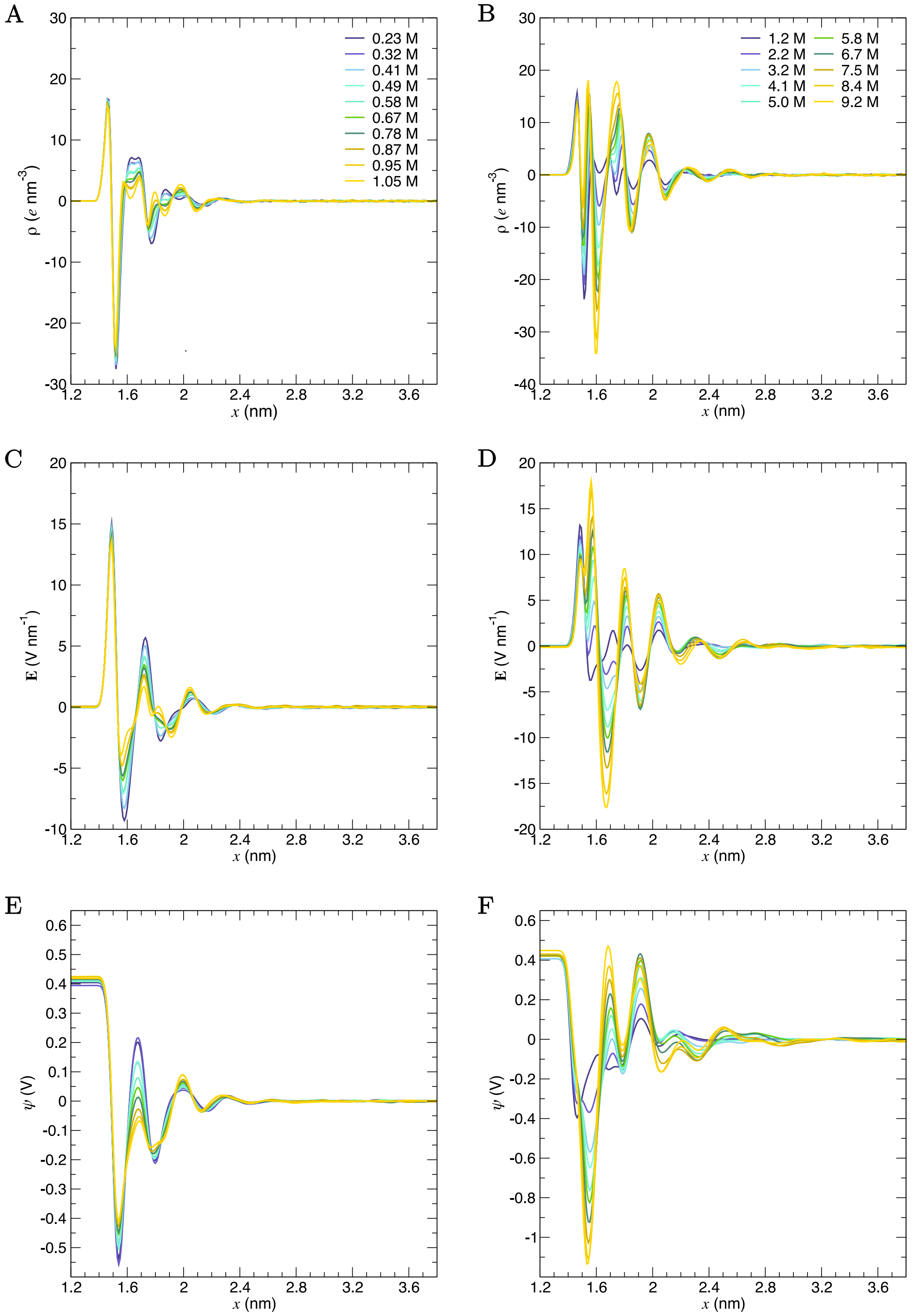

Figure S9: Solution charge densities, ρ (A and B), electric fields, E (C and D) and electric potential, ψ (E and F), as a function of distance from the centre of the simulation cell, x, in CμMD simulations with varying bulk NaCl concentrations shown by the legends in A (for A, C and D) and B (for B, D and F).

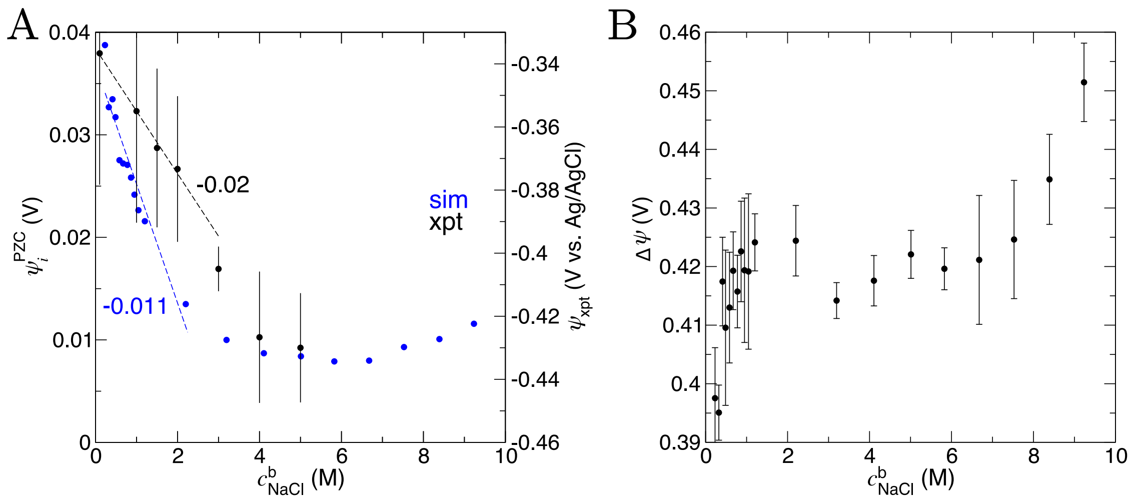

Figure S10: A: The potential difference across the double layer, ψ_i^{PZC}, as a function of the bulk concentration of ions, $c^{\mathrm{b}}_{\mathrm{NaCl}}$, evaluated in simulations using Equation 3 in the main paper when $\varepsilon_r^{\perp}(x) = 71$ (blue data). The left y-axis scale applies to the simulation data only and measurements from electrochemical impedance spectroscopy are provided by the black circles, where the right y-axis scale applies. Statistical uncertainties of one standard deviation in the data are shown by the error bars. The dashed lines are a linear fit to the data when $c^{\mathrm{b}}_{\mathrm{NaCl}} < 3$ M, the gradients for which are provided in the figure. B: The electric potential difference across the double layer, $\Delta\psi$, as a function of $c^{\mathrm{b}}_{\mathrm{NaCl}}$ evaluated when all solution species are included in the calculation of ψ using Poisson's Equation (see Equation 6 in SI Section B). $\Delta\psi$ relate to the total change in $\psi(x)$ profiles shown in panels E and F in Figure S9. Error bars highlight the standard error of the mean taken from 10 ns windows over the final 50 ns of simulation.

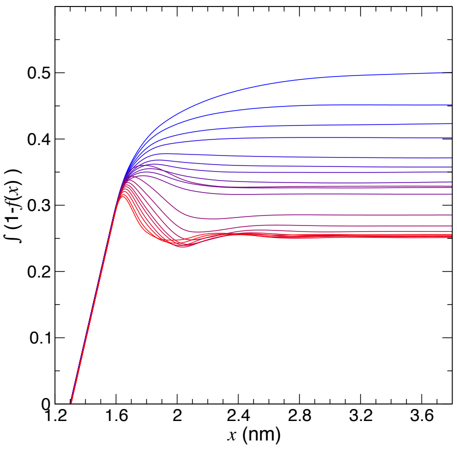

Figure S11: The cumulative integral of $(1 - f)$—with f being the screening factor presented in Figure 3 of the main paper—as a function of x. The colour scale from blue to red indicates increasing bulk ion concentrations, $c^{\mathrm{b}}_{\mathrm{NaCl}}$, over the entire sampled concentration range.

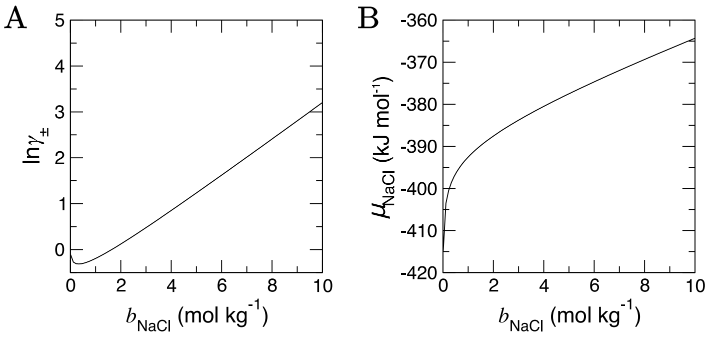

Figure S12: Mean ion activity coefficient, $\gamma_{\pm}$, and chemical potential of ions in solution, μ_{NaCl}, calculated for a range of electrolyte molalities, b_{NaCl}, using the model Equations 7 and 8 in the main paper.

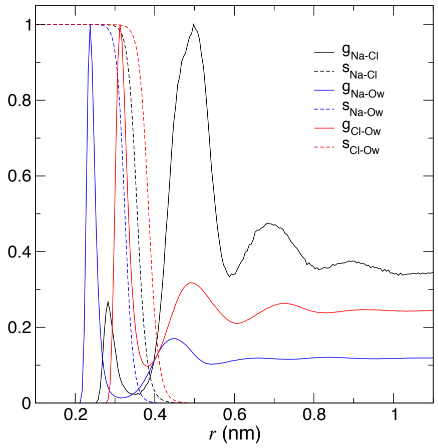

Figure S13: Pairwise radial distribution functions (RDFs), g, for atom types shown by the subscript labels in the figure key, calculated from a 10 ns bulk 1 M NaCl(aq) solution simulation. RDFs were normalised so that the maximum value is one. Also shown by the dashed lines are the switching functions, s_{ij}, used by the Plumed package to determine atoms coordinated in their respective first spheres. $s_{ij} = \frac{1-\left(\frac{r_{ij}}{r_0}\right)^{32}}{1-\left(\frac{r_{ij}}{r_0}\right)^{64}}$, where r_{ij} are the distances between atoms i and j, and with r_0 parameters provided in the main text.

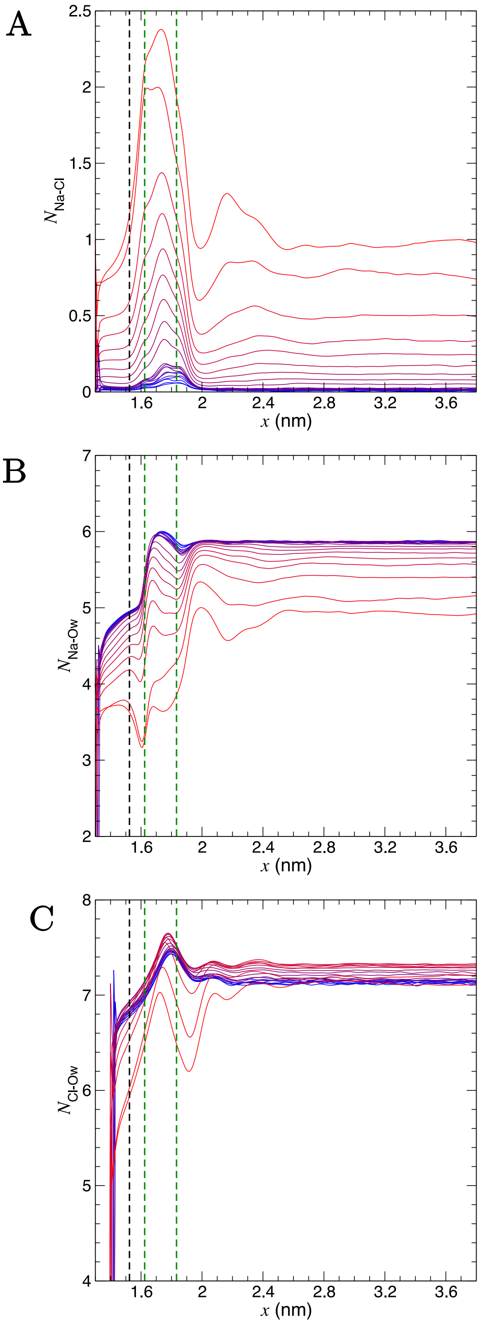

Figure S14: Average coordination numbers, N, as a function of x for Na^+ with Cl^- (A), Na^+ with O of water molecules (Ow; B) and Cl^- with O of water molecules (Ow; C). The colour scale from blue to red indicates increasing bulk ion concentrations, $c^{\mathrm{b}}_{\mathrm{NaCl}}$, across the entire concentration range sampled. The black and green dashed lines indicate the maximum first Na^+ and first two Cl^- densities in the concentration profiles for Na^+ and Cl^- highlighted in Figure 1 of the main paper. Note that the large fluctuations apparent where the distribution goes to zero are due to large statistical uncertainties in regions where the atom number densities also approach zero.

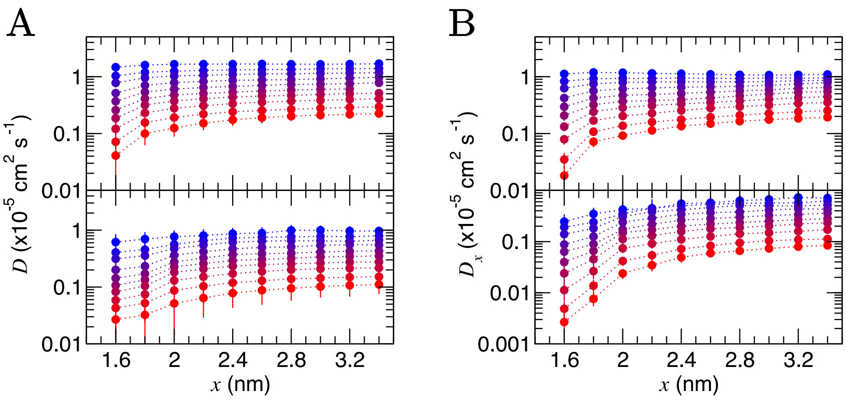

Figure S15: A: Diffusion coefficients, D, calculated in 0.4 nm regions of x moving away from the graphite surface. D is calculated from CμMD simulations with varying bulk concentrations of ions increasing from 1.2 to 9.2 M as shown by the colour scale from blue to red. B provides D_x: the x component of D. Error bars indicate uncertainties in the mean values of D and D_x from 50×1 ns trajectory windows. Top and bottom panels in A and B provide diffusion coefficients for water and ions, respectively.

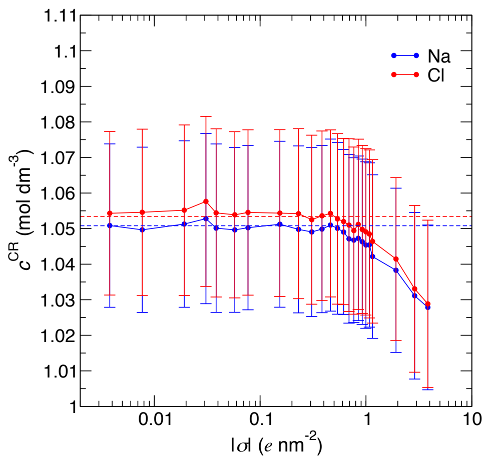

Figure S16: Mean concentrations of Na^+ and Cl^- in the control regions (c^{CR}) of CμMD simulations where the target NaCl concentration was 1 M and where surface charges, σ, were applied uniformly to the graphite surface. Error bars show the standard error in the mean values from 10 ns windows in 50 ns simulations and the dashed line provides the mean c^{CR} from simulations in the absence of graphite surface charges.

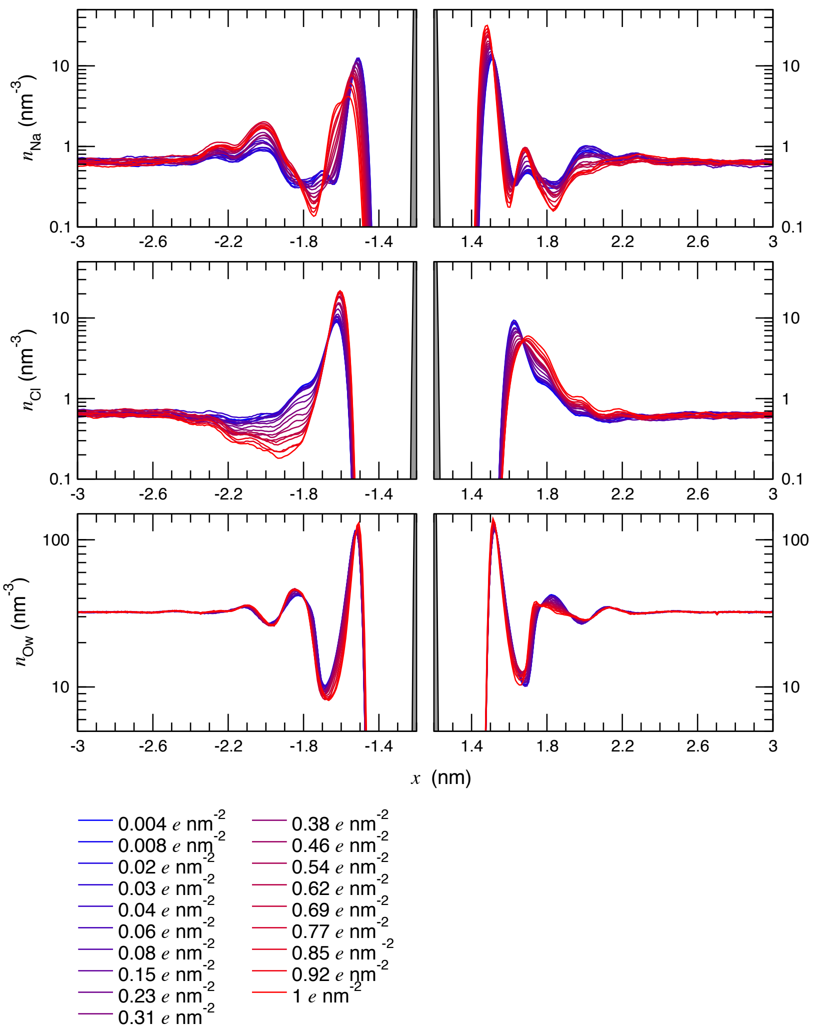

Figure S17: Number densities (n) of atom types, shown by the subscript labels (with Ow indicating water oxygen), as a function of x in CμMD simulations. Charges were applied to the graphite surface where absolute charge densities, σ, increased from 0.004 to 1 e nm^{-2}, as indicated by the blue $\rightarrow$ red colour scale and explicitly listed in the key. Left panels are the densities on a logarithmic scale, where positively charged surfaces were exposed to solution, and right panels show the corresponding solution densities at the negatively charged face of the graphite slab. The grey peaks show the position of the edge of the graphite basal surface.

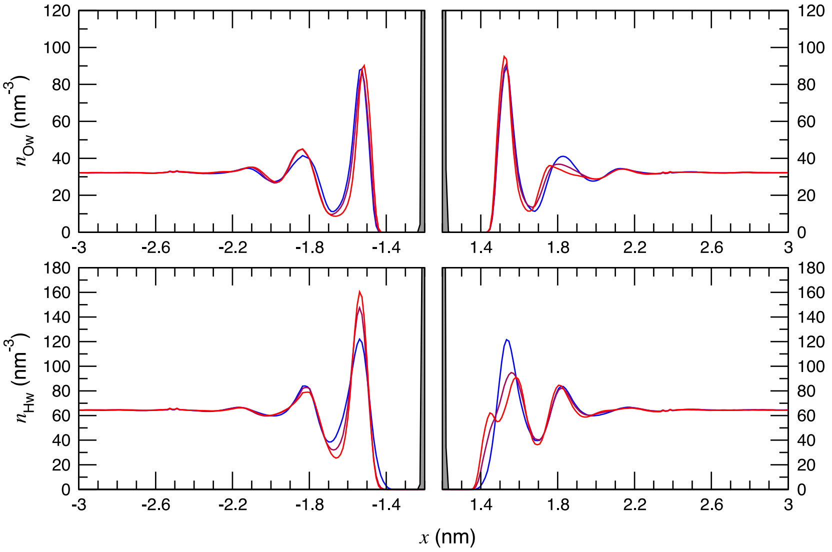

Figure S18: Water atom number densities (n) shown by the subscript labels Ow and Hw indicating water oxygen and hydrogen, respectively, from CμMD simulations with charges applied to the graphite surface. Surface charge densities, σ, were 0.004, 0.54 and 1 e nm^{-2}, indicated by the use of blue, maroon and red lines, respectively. Left panels are the densities where positively charged surfaces were exposed to solution, and right panels show the corresponding number densities at the negatively charged face of the graphite surface. The grey peaks show the position of the edge of the graphite basal slab.

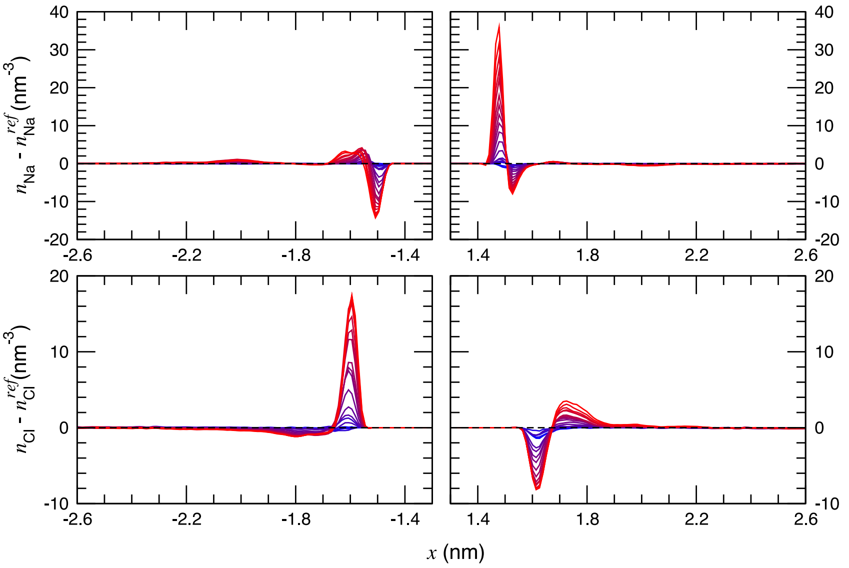

Figure S19: Excess ion number densities were calculated according to, $n - n^{ref}$, where n and n^{ref} are the ion number densities with and without applied graphite surface charges. $n - n^{ref}$ profiles in x are provided for different values of surface charge (0.004–1 e nm^{-2}) indicated by the blue → red colour scale, and the surface charge densities are explicitly listed in the key in Figure S17.

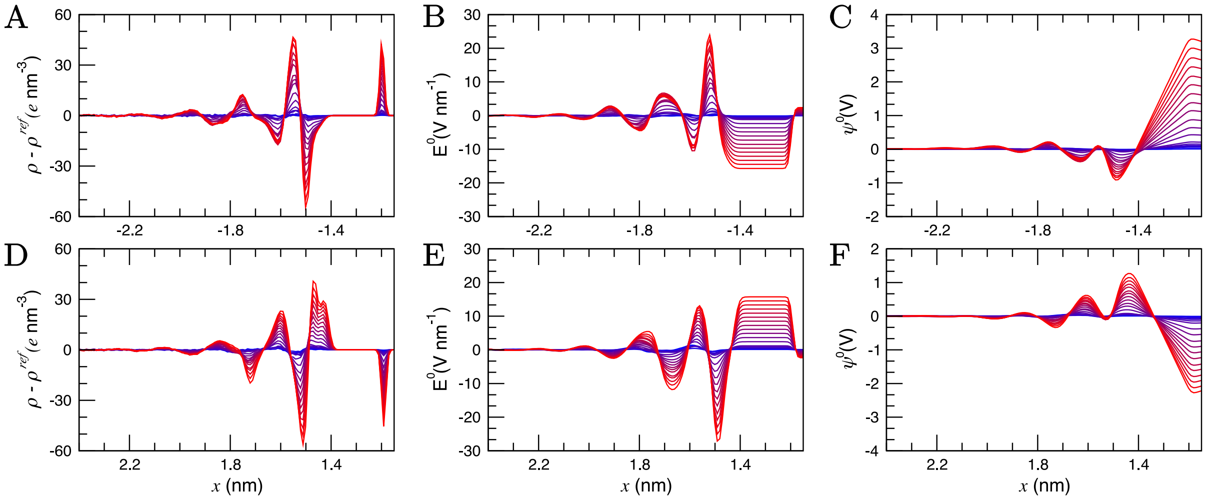

Figure S20: Excess charge density, $\rho^0 = \rho(x) - \rho^{ref}(x)$ (A,D), electric field, E^0 (B,E), and electric potential, ψ^0 (C,F), in the electric double layer calculated from CμMD simulations of NaCl(aq) solutions in contact with positively (top) and negatively (bottom) charged graphite surfaces when the charge density of the double layer in the absence of a surface charge (ρ^{ref}) was first removed. Positive and negative peaks around -1.2 and 1.2 nm in A and D, respectively, indicate the position of the graphite surface. Increasing surface charge density (in the range 0.004–1 e nm^{-2}) is indicated by the blue $\rightarrow$ red colour scale (see the key in Figure S17 for a list of the sampled charge densities).

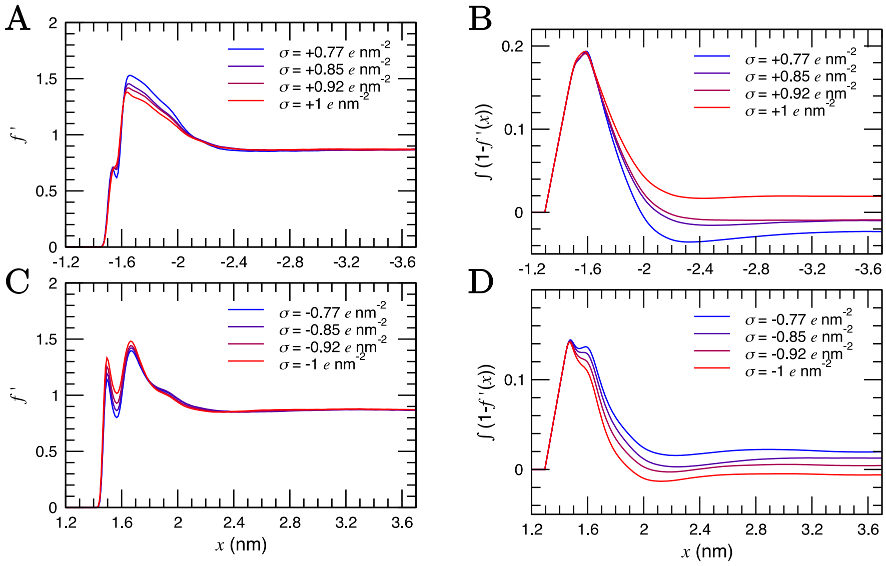

Figure S21: A and C: Electrode screening factor, f', at a positively (A) and negatively (C) charged graphite surface with surface charge density, σ, indicated by the key. B and D: The cumulative integrals of $1 - f'(x)$ using the data from A and C, respectively. The x scale in A and B (i.e. at the positively charged surface) has been reversed to ease comparison with the profiles at the negatively charged surface. Note that in A and C, f' consistently converge to a value of 0.87, i.e., lower than the predicted value of one. This is probably due to the influence of the long range contribution of the electrostatic interactions between charge carriers in solution and those on the opposite side of the graphite slab. These curves were, therefore, shifted so that $f' = 1$ at large values of x before calculating the curves in panels C and D.

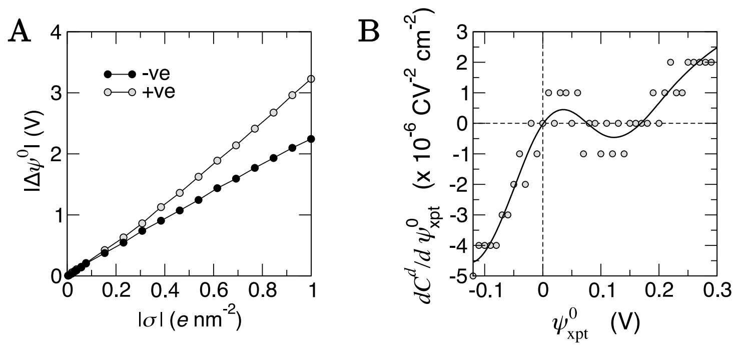

Figure S22: A: The potential difference across the double layer, $\Delta\psi^0$, when increasingly positive and negative surface charge densities, σ, were applied to the graphite surface in CμMD simulations. B: The derivative of the experimental differential capacitance, C^d, with respect to the applied potential, ψ^0_{xpt}, calculated using the data in Figure S2 for the case of 1 M. The black line is a polynomial fit to the data which highlights the trends.

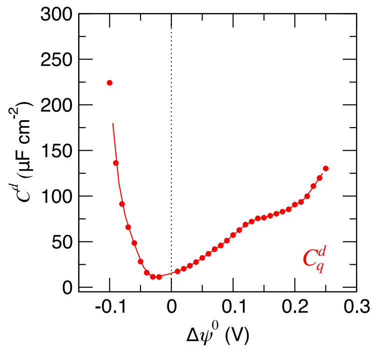

Figure S23: The 'quantum' differential capacitance (C^d_q; due to the electronic response of graphite to charging) as a function of the potential difference relative to the potential of zero charge ($\Delta\psi^0$). C^d_q was evaluated from the series $1/C^d_q = 1/C^d - 1/C^d_{dl}$ using the moving averages of simulation data (C^d_{dl}) and the experimental measurements (C^d) presented in the inset of Figure 6 F in the main paper.

References

[1] Trucano, P.; Chen, R. Structure of graphite by neutron diffraction. Nature **1975**, 258, 136.

[2] Hess, B.; Kutzner, C.; van der Spoel, D.; Lindahl, E. GROMACS 4: Algorithms for Highly Efficient, Load-Balanced, and Scalable Molecular Simulation. Journal of Chemical Theory and Computation **2008**, 4, 435–447.

[3] Bussi, G.; Donadio, D.; Parrinello, M. Canonical sampling through velocity rescaling. The Journal of Chemical Physics **2007**, 126, 014101.

[4] Berendsen, H. J. C.; Postma, J. P. M.; van Gunsteren, W. F.; DiNola, A.; Haak, J. R. Molecular dynamics with coupling to an external bath. The Journal of Chemical Physics **1984**, 81, 3684–3690.

[5] Hess, B.; Bekker, H.; Berendsen, H. J. C.; Fraaije, J. G. E. M. LINCS: A linear constraint solver for molecular simulations. Journal of Computational Chemistry **1997**, 18, 1463–1472.

[6] Essmann, U.; Perera, L.; Berkowitz, M. L.; Darden, T.; Lee, H.; Pedersen, L. G. A smooth particle mesh Ewald method. The Journal of Chemical Physics **1995**, 103, 8577–8593.

[7] Perego, C.; Salvalaglio, M.; Parrinello, M. Molecular dynamics simulations of solutions at constant chemical potential. The Journal of Chemical Physics **2015**, 142, 144113.

[8] Tribello, G. A.; Bonomi, M.; Branduardi, D.; Camilloni, C.; Bussi, G. PLUMED 2: New feathers for an old bird. Computer Physics Communications **2014**, 185, 604–613.

[9] Joung, I. S.; Cheatham, T. E. Determination of Alkali and Halide Monovalent Ion Parameters for Use in Explicitly Solvated Biomolecular Simulations. The Journal of Physical Chemistry B **2008**, 112, 9020–9041.

[10] Berendsen, H. J. C.; Grigera, J. R.; Straatsma, T. P. The missing term in effective pair potentials. The Journal of Physical Chemistry **1987**, 91, 6269–6271.

[11] Aragones, J. L.; Sanz, E.; Vega, C. Solubility of NaCl in water by molecular simulation revisited. The Journal of Chemical Physics **2012**, 136, 244508.

[12] Jorgensen, W. L.; Maxwell, D. S.; Tirado-Rives, J. Development and Testing of the OPLS All-Atom Force Field on Conformational Energetics and Properties of Organic Liquids. Journal of the American Chemical Society **1996**, 118, 11225–11236.

[13] Wu, Y.; Aluru, N. R. Graphitic Carbon–Water Nonbonded Interaction Parameters. The Journal of Physical Chemistry B **2013**, 117, 8802–8813.

[14] Ma, J.; Michaelides, A.; Alfè, D.; Schimka, L.; Kresse, G.; Wang, E. Adsorption and diffusion of water on graphene from first principles. Physical Review B **2011**, 84, 033402.

[15] Schrader, M. E. Ultrahigh vacuum techniques in the measurement of contact angles. IV. Water on graphite (0001). The Journal of Physical Chemistry **1975**, 79, 2508–2515.

[16] Prydatko, A. V.; Belyaeva, L. A.; Jiang, L.; Lima, L. M. C.; Schneider, G. F. Contact angle measurement of free-standing square-millimeter single-layer graphene. Nature Communications **2018**, 9, 4185.

[17] Li, Z.; Wang, Y.; Kozbial, A.; Shenoy, G.; Zhou, F.; McGinley, R.; Ireland, P.; Morganstein, B.; Kunkel, A.; Surwade, S. P.; Li, L.; Liu, H. Effect of airborne contaminants on the wettability of supported graphene and graphite. Nature Materials **2013**, 12, 925–931.

[18] Williams, C. D.; Dix, J.; Troisi, A.; Carbone, P. Effective Polarization in Pairwise Potentials at the Graphene–Electrolyte Interface. The Journal of Physical Chemistry Letters **2017**, 8, 703–708.

[19] Vatamanu, J.; Borodin, O.; Smith, G. D. Molecular dynamics simulations of atomically flat and nanoporous electrodes with a molten salt electrolyte. Physical Chemistry Chemical Physics **2010**, 12, 170–182.

[20] Elliott, J. D.; Troisi, A.; Carbone, P. A QM/MD Coupling Method to Model the Ion-Induced Polarization of Graphene. Journal of Chemical Theory and Computation **2020**, 16, 5253–5263.

[21] Goldsmith, Z. K.; Calegari Andrade, M. F.; Selloni, A. Effects of applied voltage on water at a gold electrode interface from *ab initio* molecular dynamics. Chemical Science **2021**, 10.1039.D1SC00354B.

[22] Tribello, G. A.; Giberti, F.; Sosso, G. C.; Salvalaglio, M.; Parrinello, M. Analyzing and Driving Cluster Formation in Atomistic Simulations. Journal of Chemical Theory and Computation **2017**, 13, 1317–1327.

[23] Yeh, I.-C.; Hummer, G. System-Size Dependence of Diffusion Coefficients and Viscosities from Molecular Dynamics Simulations with Periodic Boundary Conditions. The Journal of Physical Chemistry B **2004**, 108, 15873–15879.

[24] González, M. A.; Abascal, J. L. F. The shear viscosity of rigid water models. The Journal of Chemical Physics **2010**, 132, 096101.

[25] Joung, I. S.; Cheatham, T. E. Molecular Dynamics Simulations of the Dynamic and Energetic Properties of Alkali and Halide Ions Using Water-Model-Specific Ion Parameters. The Journal of Physical Chemistry B **2009**, 113, 13279–13290.

[26] The PLUMED consortium, Promoting transparency and reproducibility in enhanced molecular simulations. Nature Methods **2019**, 16, 670–673.

[27] Li, Z.; Wang, Y.; Kozbial, A.; Shenoy, G.; Zhou, F.; McGinley, R.; Ireland, P.; Morganstein, B.; Kunkel, A.; Surwade, S. P.; Li, L.; Liu, H. Effect of airborne contaminants on the wettability of supported graphene and graphite. Nature Materials **2013**, 12, 925–931.

[28] Hurst, J. M.; Li, L.; Liu, H. Adventitious hydrocarbons and the graphite-water interface. Carbon **2018**, 134, 464–469.

[29] Bard, A. J.; Faulkner, L. R. Electrochemical methods: fundamentals and applications, 2nd ed.; Wiley: New York, 2001.

[25] Iamprasertkun, P.; Hirunpinyopas, W.; Keerthi, A.; Wang, B.; Radha, B.; Bissett, M. A.; Dryfe, R. A. W. Capacitance of Basal Plane and Edge-Oriented Highly Ordered Pyrolytic Graphite: Specific Ion Effects. The Journal of Physical Chemistry Letters **2019**, 10, 617–623.

[31] Zou, Y.; Walton, A. S.; Kinloch, I. A.; Dryfe, R. A. W. Investigation of the Differential Capacitance of Highly Ordered Pyrolytic Graphite as a Model Material of Graphene. Langmuir **2016**, 32, 11448–11455.

[32] Uematsu, Y.; Netz, R. R.; Bonthuis, D. J. The effects of ion adsorption on the potential of zero charge and the differential capacitance of charged aqueous interfaces. Journal of Physics: Condensed Matter **2018**, 30, 064002.

[33] Helmholtz, H. Studien über electrische Grenzschichten. Annalen der Physik und Chemie **1879**, 243, 337–382.

[34] Gouy, M. Sur la constitution de la charge électrique à la surface d'un électrolyte. Journal de Physique Théorique et Appliquée **1910**, 9, 457–468.

[35] Chapman, D. L. LI. A contribution to the theory of electrocapillarity. The London, Edinburgh, and Dublin Philosophical Magazine and Journal of Science **1913**, 25, 475–481.

[36] Facci, P. Biomolecular Electronics; Elsevier, 2014; pp 19–47.

[37] Gray, C. G.; Stiles, P. J. Nonlinear electrostatics: the Poisson–Boltzmann equation. European Journal of Physics **2018**, 39, 053002.

[38] Butt, H.; Graf, K.; Kappl, M. Physics and Chemistry of Interfaces, 1st ed.; Wiley, 2003.

[39] Grahame, D. C. The Electrical Double Layer and the Theory of Electrocapillarity. Chemical Reviews **1947**, 41, 441–501.

[40] Stern, O. Zur theorie der elektrolytischen doppelschicht. Zeitschrift fur Elektrochemie und angewandte physikalische Chemie **1924**, 30, 508–516.

[41] Attard, P. Ion condensation in the electric double layer and the corresponding Poisson-Boltzmann effective surface charge. The Journal of Physical Chemistry **1995**, 99, 14174–14181.

[42] Schmickler, W. Electronic Effects in the Electric Double Layer. Chemical Reviews **1996**, 96, 3177–3200.

[43] Zhan, C.; Zhang, Y.; Cummings, P. T.; Jiang, D. Computational insight into the capacitive performance of graphene edge planes. Carbon **2017**, 116, 278–285.

[44] Fedorov, M. V.; Kornyshev, A. A. Ionic Liquids at Electrified Interfaces. Chemical Reviews **2014**, 114, 2978–3036.

[45] Dogonadze, R. R., Kálmán, E., Eds. Solvation phenomena in specific physical, chemical, and biological systems; The chemical physics of solvation ed. by Revaz R. Dogonadze; Part C; Elsevier: Amsterdam, 1988; OCLC: 256403521.

[46] Conway, B. E., Bockris, J. O., Eds. Modern Aspects of Electrochemistry: No. 13; Springer US: Boston, MA, 1979.

[47] Kornyshev, A. A. Double-Layer in Ionic Liquids: Paradigm Change? The Journal of Physical Chemistry B **2007**, 111, 5545–5557.

[48] Schlaich, A.; Knapp, E. W.; Netz, R. R. Water Dielectric Effects in Planar Confinement. Physical Review Letters **2016**, 117, 048001.

[49] Olivieri, J.-F.; Hynes, J. T.; Laage, D. Confined Water's Dielectric Constant Reduction Is Due to the Surrounding Low Dielectric Media and Not to Interfacial Molecular Ordering. The Journal of Physical Chemistry Letters **2021**, 12, 4319–4326.
